# Mental health care utilization among middle-aged and older U.S. adults: Findings from the National Health Interview Survey

**DOI:** 10.1371/journal.pmen.0000744

**Published:** 2026-10-09

**Authors:** Charllote Boateng, Isaiah Osei Duah Junior, Brandy Swati Bonnah, Bikram Adhikari, Kwadwo Owusu Akuffo, Rhodalene Asantewaa Buabeng, Engmann Yorgen Carl Nii Lomotey, Xinhua Yu

**Affiliations:** 1 Division of Epidemiology, Biostatistics, and Environmental Health, School of Public Health, The University of Memphis, Memphis, Tennessee, United States of America; 2 Department of Optometry and Visual Science, College of Science, Kwame Nkrumah University of Science and Technology, Kumasi, Ghana; 3 Department of Psychology, John R. and Kathy R. Hairston College of Health and Human Sciences, North Carolina Agricultural and Technical State University, Greensboro, North Carolina, United States of America; 4 Sinclair School of Nursing, University of Missouri, Columbia, Missouri, United States of America; PLOS: Public Library of Science, UNITED KINGDOM OF GREAT BRITAIN AND NORTHERN IRELAND

## Abstract

Mental health care (MHC) utilization remains uneven among middle-aged and older adults in the United States, yet the role of comorbidity burden and other sociodemographic, socioeconomic, behavioral, and clinical domain predictors remains incompletely characterized. We analyzed pooled data from six waves (2019–2024) of the National Health Interview Survey (NHIS), comprising 128,261 adults aged ≥40 years. Comorbidity burden was the primary predictor, categorized as none, one, or ≥2 chronic conditions, while together with additional predictors was organized according to the predisposing, enabling, and need domains of Andersen’s Behavioral Model of Health Services Use. MHC utilization, the outcome, was defined as counseling or psychotherapy, mental health medication use, or visits to a mental health specialist within the previous 12 months. Survey-weighted multivariable logistic regression accounted for the complex survey design and sampling weights. Overall, approximately one in five adults utilized MHC services. Utilization was higher among females, non-Hispanic White adults, unmarried adults, smokers, overweight or obese adults, those with lower income, those with health insurance coverage, or residence in the Midwest, and those with one or ≥2 comorbidities, depression, or anxiety. In contrast, utilization was lower among adults aged 50–64, 65–74, and ≥75 years, non-Hispanic Black and Asian adults, and those with higher educational attainment. Taken together, these findings indicate that MHC utilization is shaped by comorbidity burden alongside predisposing, enabling, and need-related factors, underscoring the need for targeted and culturally responsive strategies to improve equitable access to MHC among this ageing population.

## Author summary

Many middle-aged and older adults in the U.S. live with chronic health conditions, yet some do not use mental health care (MHC) even when they may have mental health needs. We analyzed data from 128,261 adults aged 40 years and older who participated in the National Health Interview Survey from 2019 to 2024. About one in five adults used MHC, including counseling, mental health medicines, or visits to mental health professionals. We examined comorbidity burden as the main predictor of MHC use, while also considering other factors based on Andersen’s model, including personal characteristics, access to care, and health needs. MHC use was higher among women, White adults, unmarried adults, smokers, people who were overweight or obese, those with lower incomes, those with health insurance, and those living in the Midwest. Use was also higher among people with one or more chronic conditions, depression, or anxiety. In contrast, MHC use was lower among adults aged 50 years and older, Black and Asian adults, and those with higher levels of education. These findings show that both chronic health conditions and social and personal factors may influence whether middle-aged and older adults receive MHC or not. Hence, expanding affordable, accessible, and culturally appropriate services may help reduce gaps in care and connect aging adults with the mental health support they need to promote healthy ageing and mental well-being**.**

## Introduction

Mental health disorders remain a major public health concern in the United States [1–3]. Yet engagement with MHC services remains uneven across populations [4–7]. They frequently coexist with chronic physical conditions [8–10], a combination associated with poorer functional outcomes [11–13], higher healthcare costs [14,15], and increased mortality [16,17]. Despite these concerns, evidence on how middle-aged and older adults with comorbidities, particularly those with one or multiple chronic conditions, engage with MHC services remains limited. Previous studies have examined demographic, socioeconomic, behavioral, and clinical factors associated with MHC utilization in the general population [6,18,19], but patterns of MHC use among adults with comorbidities and the factors associated with such use remain incompletely characterized.

The Andersen Behavioral Model of Health Services Use provides a useful framework for organizing factors that may influence healthcare utilization across three domains: predisposing characteristics, enabling resources, and need factors [20–24]. Predisposing characteristics include demographic attributes such as age, sex, and race/ethnicity; enabling factors include socioeconomic and healthcare-related resources such as income, education, insurance coverage, and geographic access; and need factors include health status, comorbidity, and perceived or evaluated mental health needs [20–24]. In the present study, this framework serves primarily as an organizing model for examining the range of factors associated with MHC utilization rather than as a formal test of the model. This approach allows sociodemographic, socioeconomic, behavioral, and clinical characteristics to be considered within a common conceptual structure while recognizing that these domains may interact in shaping access to and use of MHC services.

Prior research has identified disparities in MHC utilization across sex, age, race/ethnicity, and geographic region [25,26]. Historically underserved populations, including Black/African American and Asian adults, have reported lower utilization of MHC services than other racial/ethnic groups [27–29]. Socioeconomic and healthcare-related resources may also influence access to care, with education, income, and insurance coverage shaping the ability to obtain and sustain MHC services [20–24]. Clinical and behavioral characteristics may further influence both perceived need for care and help-seeking behavior [30–32]. Comorbidity is particularly relevant because ageing adults with chronic physical conditions may experience greater psychological burden, more complex healthcare needs, and increased opportunities for interaction with the healthcare system [31,33–36]. However, relatively few studies have simultaneously examined these predisposing, enabling, and need-related factors among middle-aged and older adults with comorbidities at the national level [6,25,37–40].

Given the complex needs of these adults with comorbidities, we hypothesized that MHC utilization would be higher among adults with greater comorbidity burden and would vary according to predisposing, enabling, and need-related factors, including sociodemographic characteristics, socioeconomic resources, and clinical and behavioral factors. The present study used nationally representative data from U.S. adults aged ≥40 years to examine patterns of mental health care utilization and the relationships between comorbidity burden and service use, while accounting for sociodemographic, behavioral, clinical, and enabling factors. Guided by the Andersen Behavioral Model as an organizing framework, we examined predisposing factors (age, sex, race/ethnicity, and marital status), enabling factors (education, income, health insurance, and geographic region), and need-related and health-related factors (comorbidity burden, depression, anxiety, smoking, and body mass index). By examining these factors collectively, this study aimed to identify populations with greater or lower engagement in MHC services and provide evidence to inform targeted interventions and policies aimed at improving equitable access to mental healthcare in the United States.

## Materials and methods

### Ethical statement

This study utilized secondary data from the NHIS, a publicly available dataset with all personally identifiable information removed before release. As no direct contact was made with participants and no new data were collected, the study posed minimal risk to individuals. The analysis adhered to established ethical guidelines for the use of de-identified secondary data, and institutional review board (IRB) approval was not applicable. All procedures followed the principles of confidentiality and respect for participants as outlined in federal human subject protections and adhered to the Tenets of the Declaration of Helsinki.

### Study design and population

This cross-sectional study used nationally representative data from six consecutive waves (2019–2024) of the NHIS, which collects information on health status, health care utilization, and health behaviors among the U.S. civilian, non-institutionalized population. The NHIS uses a complex multistage probability sampling design with stratification and clustering to generate nationally representative estimates. For the present study, the pooled dataset was restricted a priori to adults aged ≥40 years**,** representing middle-aged and older adults. The age-restricted pooled sample included 130,312 participants (2019: n = 22,345; 2020: n = 23,029; 2021: n = 20,886; 2022: n = 19,706; 2023: n = 21,073; 2024: n = 23,273). After harmonizing variables across survey waves, 2,051 participants (1.6%) with missing information on the primary outcome, MHC utilization during the past 12 months, were excluded**,** yielding a final sample of 128,261 adults with complete MHC utilization data**.** This sample was used for descriptive analyses. For the multivariable regression analyses, an additional 7,229 participants were excluded because of missing information on one or more model covariates**,** resulting in a complete-case analytic sample of 121,032 adults**.** Missing covariate information included BMI (*n* = 3,056; 2.4%), marital status (*n* = 2,451; 1.9%), education (*n* = 1,814; 1.4%), smoking status (*n* = 1,389; 1.1%), and sex (*n* = 6; < 0.1%) as shown in Fig 1. The ≥ 40-year cutoff was selected to focus on middle-aged and older adults, among whom the burden of chronic conditions and related health care needs increases with age. All analyses incorporated NHIS sampling weights, primary sampling units, and strata to account for the complex survey design and generate nationally representative estimates with appropriate variance estimation.

**Fig 1:**
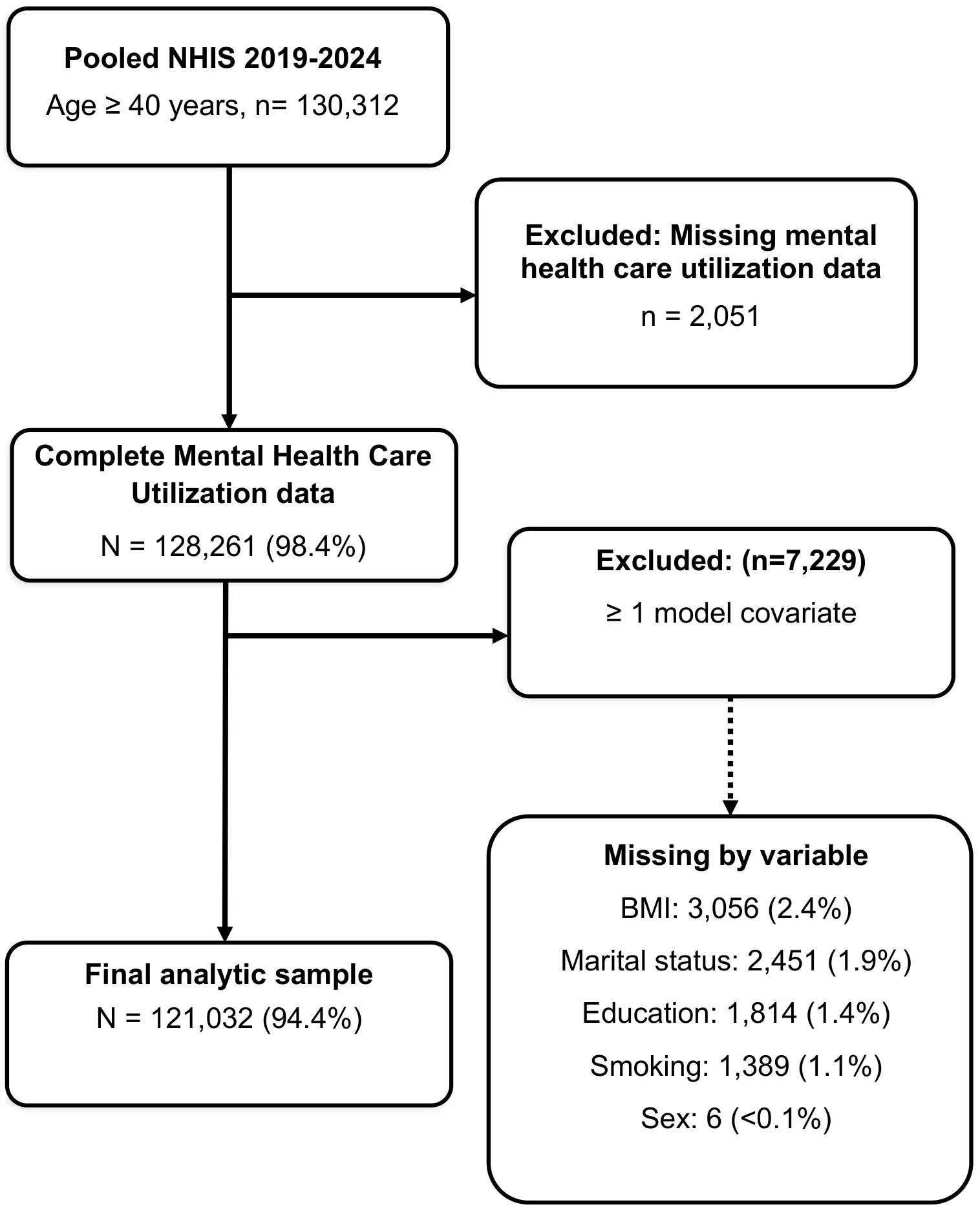
Study flow diagram. This diagram depicts the initial pooled study population and the sequential application of exclusion criteria that resulted in the final analytical sample.

### Measures

The primary outcome was MHC service utilization, defined as a binary indicator of any mental health service use in the past 12 months, including counseling or psychotherapy, prescription medication for mental health conditions, or visits to a mental health specialist.

The primary predictor was comorbidity burden, categorized as none, one, or ≥2 chronic conditions (multimorbidity), based on self-reported physician diagnoses. Six chronic conditions consistently available across all survey years were included: hypertension, diabetes, arthritis, cancer, asthma, and coronary heart disease. Additional predictors were classified according to Andersen’s Behavioral Model of Health Services Use. Predisposing factors included sex, age, race/ethnicity, marital status, smoking status, and BMI; enabling factors included education, ratio of family income to the poverty threshold, income level, health insurance coverage, urban-rural residence, and U.S. region; and need factors included comorbidity burden, depression, and anxiety. Depression and anxiety were based on whether respondents had ever been told by a doctor or other health professional that they had these conditions, rather than on symptom frequency, severity, or screening-based assessments. Given substantial missingness in the specific type of insurance coverage, this variable was excluded from the analyses. Altogether, adoption of this approach distinguished pre-existing, self-reported mental health conditions from the MHC utilization outcome, which captured mental health treatment received during the preceding 12 months.

### Statistical analysis

A systematic analytic approach was applied. First, both unweighted and survey-weighted descriptive statistics (frequencies and percentages) were used to characterize the study population and patterns of MHC utilization. Unweighted estimates described the observed analytic sample, whereas survey-weighted estimates accounted for the complex sampling design and provided nationally representative population estimates. Second, differences across groups were assessed using design-adjusted chi-square tests for categorical variables and appropriate survey-adjusted tests for continuous variables. Third, survey-weighted logistic regression models were fitted to estimate odds ratios (ORs) and 95% confidence intervals (CIs) for associations between study covariates and MHC utilization. Univariate logistic regression models were initially fitted to examine the association between each predictor and MHC utilization. Variables demonstrating a statistically significant overall association (*p* ≤ 0.05) were selected for inclusion in the multivariable model, with consideration of their theoretical relevance to MHC utilization. For categorical variables selected for the multivariable model, all constituent categories were retained regardless of the statistical significance of individual category-level coefficients, with one category designated as the reference group. This approach ensured that categorical predictors were retained as complete variables rather than selectively excluding individual levels based on category-specific *p*-values. The multivariable models therefore estimated adjusted associations while accounting for potential confounding among the selected predictors. Before fitting the multivariable models, multicollinearity among the candidate covariates was assessed using variance inflation factors (VIFs)**.** VIFs were examined for the predictors included in the regression models, with higher values indicating greater linear dependence among covariates. The diagnostic assessment did not identify problematic multicollinearity that would compromise model estimation or interpretation (see S1 Table). Complete-case analysis was used to address missing data, with missingness below 2.5% across the variables included in the analyses. Sensitivity analyses compared unweighted and survey-weighted estimates and evaluated alternative categorizations of key variables to assess the robustness of the findings. All survey-weighted analyses incorporated sampling weights, strata, and primary sampling units to account for the complex survey design and support population-level inference. Finally, forest plots, bar graphs, and line graphs were used to complement the regression and descriptive analyses. Forest plots presented adjusted odds ratios (AORs) and 95% confidence intervals from the multivariable logistic regression models, facilitating comparison of the direction, magnitude, and precision of associations across predictors. Bar graphs were used to visually summarize distributions and differences in MHC utilization across key sociodemographic, socioeconomic, and health-related characteristics. Line graphs were used to illustrate patterns and trends in MHC utilization across survey years. These graphical presentations complemented the tabular results and facilitated interpretation of the observed patterns and regression estimates. Analyses were performed in R version 4.4.2 using survey-specific procedures.

## Results

### Distribution of MHC utilization among middle-aged and older U.S. adults with comorbidities

Table 1 presents patterns of MHC utilization among 128,261 U.S. adults, of whom 28,396 (22.1%) reported MHC utilization. Utilization differed significantly across all sociodemographic, socioeconomic, and health-related characteristics examined (all p < 0.001). MHC users were predominantly female (67.6% vs. 51.4% among non-users) and were slightly younger (median age, 61 vs. 63 years), with a greater proportion aged 50–64 years (37.6% vs. 34.8%) and fewer aged ≥75 years (15.9% vs. 20.5%). The distribution by race and ethnicity also differed, with MHC users more frequently identifying as non-Hispanic White (80.4% vs. 69.5%) and less frequently as Hispanic (8.0% vs. 11.7%), non-Hispanic Black (7.5% vs. 11.2%), or non-Hispanic Asian (1.8% vs. 5.6%). Socioeconomic differences were also evident. Although educational attainment was broadly similar between groups, MHC users had a lower median family income-to-poverty ratio (3.2 vs. 3.7) and a greater proportion lived below the poverty threshold (13.1% vs. 8.2%) or had low income (19.2% vs. 16.9%), while fewer had high income (39.7% vs. 45.4%). MHC users were also less likely to be married (44.3% vs. 52.7%) and more likely to report neither being married nor living with a partner (51.6% vs. 43.4%). They were more concentrated in medium or small metropolitan areas (32.5% vs. 30.8%) and non-metropolitan areas (19.3% vs. 17.6%), although regional distributions were broadly comparable. Nearly all MHC users had health insurance coverage (97.2% vs. 93.9%). Health-related differences were particularly pronounced: MHC users had a higher prevalence of multimorbidity, with 54.4% reporting two or more comorbid conditions compared with 38.8% of non-users, and lower proportions reporting no comorbid conditions (19.3% vs. 31.6%). They were also more likely to smoke every day (13.1% vs. 8.8%), have obesity (39.7% vs. 31.9%), and report depression (29.2% vs. 4.3%) or anxiety (51.9% vs. 14.8%), see Table 1. The survey-weighted characteristics of the study population (S2 Table). All descriptive estimates incorporated NHIS survey weights, strata, and primary sampling units to account for the complex multistage sampling design and to obtain nationally representative estimates of the U.S. civilian, noninstitutionalized adult population. The overall weighted distribution of MHC utilization across sociodemographic, socioeconomic, behavioral, and health characteristics was consistent with the unweighted sample, with no substantial differences in the distribution of key study variables (see S2 Table).

**Table 1:** Description of the analytical sample.

Variables	Total (n = 128,261,100%)^1^	Yes (n = 28,396, 22.1%)	No (n = 99,865, 77.9%)	p-value
**Sociodemographic characteristics**				
**Sex** ^**Φ**^				<0.001
Male	57,785 (45.1)	9,207 (32.4)	48,578 (48.6)	
Female	70,470 (54.9)	19,188 (67.6)	51,282 (51.4)	
Unknown	6	1	5	
**Age in years** ^**Φ**^	62.0 (52.0, 72.0)	61.0 (51.0, 70.0)	63.0 (52.0, 72.0)	<0.001
**Age category in years** ^**Φ**^				<0.001
40-49	25,834 (20.1)	6,323 (22.3)	19,511 (19.5)	
50-64	45,381 (35.4)	10,663 (37.6)	34,718 (34.8)	
65-74	32,073 (25.0)	6,899 (24.3)	25,174 (25.2)	
≥ 75 (75+)	24,973 (19.5)	4,511 (15.9)	20,462 (20.5)	
**Race** ^**Φ**^				<0.001
Hispanic	13,953 (10.9)	2,271 (8.0)	11,682 (11.7)	
Non-Hispanic White	92,235 (71.9)	22,844 (80.4)	69,391 (69.5)	
Non-Hispanic Black	13,282 (10.4)	2,128 (7.5)	11,154 (11.2)	
Non-Hispanic Asian	6,089 (4.7)	513 (1.8)	5,576 (5.6)	
Non-Hispanic AIAN	828 (0.6)	175 (0.6)	653 (0.7)	
Non-Hispanic AIAN, Multiple Races	952 (0.7)	306 (1.1)	646 (0.6)	
Non-Hispanic Other/Multiple Races	922 (0.7)	159 (0.6)	763 (0.8)	
**Educational category** ^**δ**^				<0.001
Less than high school	11,660 (9.2)	2,371 (8.5)	9,289 (9.4)	
High school graduate/GED	51,099 (40.4)	11,125 (39.7)	39,974 (40.6)	
Some college/Associate	17,407 (13.8)	4,156 (14.8)	13,251 (13.5)	
Bachelor’s or higher	46,281 (36.6)	10,363 (37.0)	35,918 (36.5)	
Unknown	1,814	381	1,433	
**Marital status** ^**δ**^				<0.001
Married	63,929 (50.8)	12,375 (44.3)	51,554 (52.7)	
Living with partner (unmarried)	4,963 (3.9)	1,148 (4.1)	3,815 (3.9)	
Neither	56,918 (45.2)	14,405 (51.6)	42,513 (43.4)	
Unknown	2,451	468	1,983	
**Urban-rural residence** ^**δ**^				<0.001
Large Metro	35,120 (27.4)	7,223 (25.4)	27,897 (27.9)	
Large Fringe Metro	30,079 (23.5)	6,444 (22.7)	23,635 (23.7)	
Medium and Small Metro	39,946 (31.1)	9,235 (32.5)	30,711 (30.8)	
Non-metropolitan	23,116 (18.0)	5,494 (19.3)	17,622 (17.6)	
**Region** ^**δ**^				<0.001
Northeast	21,739 (16.9)	4,904 (17.3)	16,835 (16.9)	
Midwest	28,778 (22.4)	6,961 (24.5)	21,817 (21.8)	
South	46,577 (36.3)	10,197 (35.9)	36,380 (36.4)	
West	31,167 (24.3)	6,334 (22.3)	24,833 (24.9)	
***Socioeconomic characteristics***				
**Ratio of family income to poverty threshold** ^**δ**^	3.5 (1.9, 5.9)	3.2 (1.6, 5.6)	3.7 (2.0, 6.1)	<0.001
**Income level** ^**δ**^				<0.001
Below poverty	11,947 (9.3)	3,732 (13.1)	8,215 (8.2)	
Low income	22,330 (17.4)	5,443 (19.2)	16,887 (16.9)	
Moderate	37,359 (29.1)	7,935 (27.9)	29,424 (29.5)	
High	56,625 (44.1)	11,286 (39.7)	45,339 (45.4)	
**Insurance coverage** ^**δ**^				<0.001
Uninsured	6,886 (5.4)	799 (2.8)	6,087 (6.1)	
Insured	121,375 (94.6)	27,597 (97.2)	93,778 (93.9)	
***Behavioral and health characteristics***				
**Comorbidity category** ^**τ**^				<0.001
No comorbid	37,040 (28.9)	5,478 (19.3)	31,562 (31.6)	
One comorbid	37,023 (28.9)	7,481 (26.3)	29,542 (29.6)	
Two or more comorbid	54,198 (42.3)	15,437 (54.4)	38,761 (38.8)	
**Smoking status** ^**Φ**^				<0.001
Not at all	111,357 (87.8)	23,536 (83.7)	87,821 (88.9)	
Somedays	3,181 (2.5)	898 (3.2)	2,283 (2.3)	
Everyday	12,334 (9.7)	3,676 (13.1)	8,658 (8.8)	
Unknown	1,389	286	1,103	
**Body Mass Index (BMI)** ^**Φ**^				<0.001
Underweight	1,772 (1.4)	386 (1.4)	1,386 (1.4)	
Healthy weight	36,415 (29.1)	7,357 (26.5)	29,058 (29.8)	
Overweight	44,941 (35.9)	9,003 (32.4)	35,938 (36.9)	
Obese	42,077 (33.6)	11,026 (39.7)	31,051 (31.9)	
Unknown	3,056	624	2,432	
**Depression** ^**τ**^				<0.001
No	115,670 (90.2)	20,109 (70.8)	95,561 (95.7)	
Yes	12,591 (9.8)	8,287 (29.2)	4,304 (4.3)	
**Anxiety** ^**τ**^				<0.001
No	98,732 (77.0)	13,653 (48.1)	85,079 (85.2)	
Yes	29,529 (23.0)	14,743 (51.9)	14,786 (14.8)	

Unweighted frequencies and percentages; Yes, utilize MHC service; No, not utilize MHC service; n (%); Median (Q1, Q3); Pearson’s Chi-squared tests; Wilcoxon rank sum test; N is not equal to 128,261 for all variables distributed across MHC utilization; AIAN: American Indian and Alaska Natives; ^**Φ**^**Predisposing factors** (Sex, age, race/ethnicity, marital status, smoking status, BMI.);^**δ**^ **Enabling factors** (Education, ratio of family income to poverty threshold, income level, insurance coverage, urban-rural residence, region);^**τ**^ **Need factors (**Comorbidity, depression, anxiety) based on Andersen’s Model of Health Utilization.

### Trends in mental health care utilization by age group and comorbidity status

MHC utilization was consistently highest among adults with two or more comorbidities, followed by those with one comorbidity, across all age groups and survey years. Utilization generally increased over time among adults aged 40–49 and 50–64, while patterns were relatively stable among those aged 65–74. Among adults aged ≥75 years, utilization remained highest in the multimorbidity group but showed greater year-to-year variation, particularly among those without comorbidity (see Fig 2).

**Fig 2:**
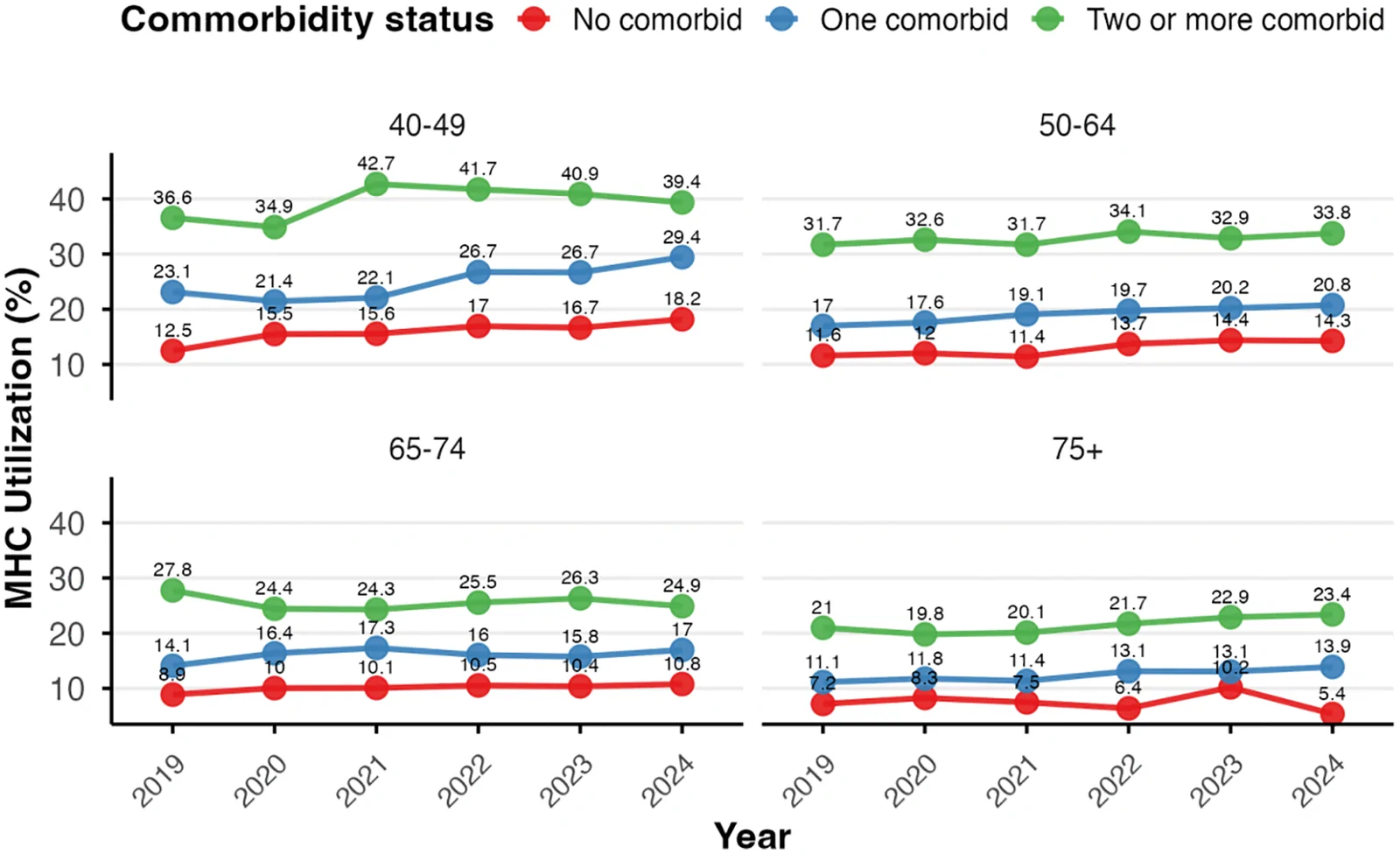
Trends in MHC utilization by age group and comorbidity status. Percent estimates represent the proportion of participants with MHC utilization in each year, with categories summing to 100% within each year.

### Factors related to MHC service utilization among U.S. middle-aged and older adults

For the primary model with depression and anxiety (see Table 2), the univariate logistic regression showed that female sex was related to nearly twice the odds of MHC utilization compared with males (COR = 1.99, 95% CI: 1.92–2.07; p < 0.001). Compared with adults aged 40–49 years, those aged 65–74 and ≥75 years had lower odds of MHC utilization (COR = 0.89, 95% CI: 0.84–0.94; p < 0.001 and COR = 0.77, 95% CI: 0.73–0.82; p < 0.001, respectively). Non-Hispanic Whites had higher odds of MHC utilization than Hispanics (COR = 1.94, 95% CI: 1.83–2.06; p < 0.001), whereas non-Hispanic Asians had lower odds (COR = 0.48, 95% CI: 0.42–0.55; p < 0.001). Compared with adults with less than high school, those with High school graduate/GED (COR = 1.19, 95% CI: 1.12–1.26; p < 0.001), Some college or associate (COR = 1.34, 95% CI: 1.25–1.44; p < 0.001), and bachelor’s degree or highest (COR = 1.23, 95% CI: 1.16–1.31; p < 0.001) were related with higher odds of MHC utilization. Relative to married participants, those living with a partner together as an unmarried couple (COR = 1.23, 95% CI: 1.13–1.34; p < 0.001) and those single, i.e., neither married (COR = 1.47, 95% CI: 1.42–1.52; p < 0.001), were related to higher odds of MHC utilization. For urban–rural residence, compared with ageing adults living in large metropolitan areas, those residing in large fringe metropolitan, medium or small metropolitan, and non-metropolitan areas had 9%, 22%, and 31% higher odds of MHC utilization, respectively (COR = 1.09, 95% CI: 1.03–1.15; p = 0.004; COR = 1.22, 95% CI: 1.15–1.30; p < 0.001; and COR = 1.31, 95% CI: 1.22–1.41; p < 0.001). Compared with ageing adults residing in the Northeast, those in the Midwest had 18% higher odds of MHC utilization (COR = 1.18, 95% CI: 1.10–1.26; p < 0.001), whereas those in the West had 11% lower odds (COR = 0.89, 95% CI: 0.83–0.97; p = 0.006). Higher comorbidity was strongly related to MHC utilization, with COR of 1.50 (95% CI: 1.43–1.57; p < 0.001) for one comorbidity and 2.51 (95% CI: 2.40–2.63; p < 0.001) for ≥2 comorbidities. Compared with ageing adults who did not smoke, those who smoked on some days and every day had 37% and 62% higher odds of MHC utilization, respectively (COR = 1.37, 95% CI: 1.25–1.50; p *<* 0.001; and COR = 1.62, 95% CI: 1.53–1.70; p *<* 0.001). Unlike adults with normal weight, those who were obese had 38% higher odds of MHC utilization (COR = 1.38, 95% CI: 1.32–1.44; p < 0.001). Compared with high-income adults, those with low income and those living below the poverty line had 21% and 59% higher odds of MHC utilization, respectively (COR = 1.21, 95% CI: 1.16–1.28; p *<* 0.001; and COR = 1.59, 95% CI: 1.50–1.68; p *<* 0.001). Insured participants had higher odds of MHC utilization than uninsured participants (COR = 2.43, 95% CI: 2.22–2.66; p < 0.001). The strongest crude relationships were observed for depression (COR = 9.66, 95% CI: 9.18–10.16; p < 0.001) and anxiety (COR = 6.38, 95% CI: 6.15–6.61; p < 0.001).

**Table 2:** Factors associated with MHC utilization among middle-aged and older U.S. adults with comorbidities.

Variable	Univariate Logistic Regression			Multivariable Logistic Regression		
Model statistics	COR	95%CI	p-value	AOR	95%CI	p-value
**Sex** ^**Φ**^						
Male	Ref			Ref		
Female	1.99	1.92 – 2.07	<0.001	1.95	1.87 – 2.04	<0.001
**Age category in years** ^**Φ**^						
40-49	Ref			Ref		
50-64	0.97	0.93 – 1.01	0.158	0.80	0.76 – 0.84	<0.001
65-74	0.89	0.84 – 0.94	<0.001	0.65	0.61 – 0.69	<0.001
≥ 75 (75+)	0.77	0.73 – 0.82	<0.001	0.51	0.47 – 0.54	<0.001
**Race** ^**Φ**^						
Hispanic	Ref			Ref		
Non-Hispanic White	1.94	1.83 – 2.06	<0.001	1.70	1.58 – 1.82	<0.001
Non-Hispanic Black	1.09	0.99 – 1.19	0.071	0.81	0.74 – 0.89	<0.001
Non-Hispanic Asian	0.48	0.42 – 0.55	<0.001	0.54	0.47 – 0.62	<0.001
Non-Hispanic AIAN	1.54	1.05 – 2.26	0.029	0.97	0.66 – 1.44	0.893
Non-Hispanic AIAN, Multiple Races	2.53	2.12 – 3.03	<0.001	1.52	1.21 – 1.91	<0.001
Non-Hispanic Other/Multiple Races	1.27	1.00 – 1.60	0.048	0.92	0.71 – 1.18	0.495
**Educational category** ^**Φ**^						
Less than high school	Ref			Ref		
High school graduate/GED	1.19	1.12 – 1.26	<0.001	0.70	0.64 – 0.76	<0.001
Some college/Associate	1.34	1.25 – 1.44	<0.001	0.79	0.75 – 0.83	<0.001
Bachelor’s or higher	1.23	1.16 – 1.31	<0.001	0.88	0.83 – 0.93	<0.001
**Marital status** ^**δ**^						
Married	Ref			Ref		
Living with partner together as unmarried couple	1.23	1.13 – 1.34	<0.001	1.06	0.96 – 1.16	0.255
Neither	1.47	1.42 – 1.52	<0.001	1.23	1.18 – 1.28	<0.001
**Urban-rural residence** ^**δ**^						
Large Metro	Ref			Ref		
Large Fringe Metro	1.09	1.03 – 1.15	0.004	0.97	0.90 – 1.02	0.243
Medium and Small Metro	1.22	1.15 – 1.30	<0.001	0.99	0.94 – 1.05	0.761
Non-metropolitan	1.31	1.22 – 1.41	<0.001	0.95	0.88 – 1.02	0.157
**Region** ^**δ**^						
Northeast	Ref			Ref		
Midwest	1.18	1.10 – 1.26	<0.001	1.10	1.03 – 1.17	0.006
South	1.00	0.94 – 1.08	0.927	1.04	0.98 – 1.10	0.225
West	0.89	0.83 – 0.97	0.006	0.97	0.90– 1.03	0.326
**Comorbidity category** ^**τ**^						
No comorbid	Ref			Ref		
One comorbid	1.50	1.43 – 1.57	<0.001	1.42	1.35 – 1.49	<0.001
Two or more comorbid	2.51	2.40 – 2.63	<0.001	2.23	2.11 – 2.34	<0.001
**Smoking status** ^**Φ**^						
Not at all	Ref			Ref		
Somedays	1.37	1.25 – 1.50	<0.001	1.23	1.10 – 1.37	<0.001
Everyday	1.62	1.53 – 1.70	<0.001	1.12	1.05 – 1.20	0.001
**Body Mass Index (BMI)** ^**Φ**^						
Normal	Ref			Ref		
Underweight	1.14	1.00 – 1.31	0.054	0.85	0.72 – 1.00	0.050
Overweight	0.97	0.94 – 1.01	0.201	1.09	1.04 – 1.15	<0.001
Obese	1.38	1.32 – 1.44	<0.001	1.23	1.17 – 1.29	<0.001
**Income level** ^**δ**^						
High	Ref			Ref		
Moderate	1.04	1.00 – 1.08	0.086	0.97	0.93 – 1.02	0.268
Low income	1.21	1.16 – 1.28	<0.001	1.08	1.02 – 1.15	0.010
Below poverty	1.59	1.50 – 1.68	<0.001	1.23	1.14 – 1.33	<0.001
**Insurance coverage** ^**δ**^						
Uninsured	Ref			Ref		
Insured	2.43	2.22 – 2.66	<0.001	2.51	2.24 – 3.83	<0.001
**Depression** ^**τ**^						
No	Ref			Ref		
Yes	9.66	9.18 – 10.16	<0.001	4.08	3.83 – 4.34	<0.001
**Anxiety** ^**τ**^						
No	Ref			Ref		
Yes	6.38	6.15 – 6.61	<0.001	3.49	3.35 – 3.64	<0.001

Complete observations for regression analysis : 121,032; ^**Φ**^**Predisposing factors** (Sex, age, race/ethnicity, marital status, smoking status, BMI);^**δ**^ **Enabling factors** (Education, ratio of family income to poverty threshold, income level, insurance coverage, urban-rural residence, region);^**τ**^ **Need factors** (Comorbidity, depression, anxiety) based on Andersen’s Model of Health Utilization. Univariate logistic regression analyses were initially conducted to examine the relationship between each predictor and MHC utilization. Variables demonstrating a statistically significant overall relationship with MHC utilization (*p* ≤ 0.05) were selected for inclusion in the multivariable logistic regression model. For categorical predictors selected for multivariable analysis, all constituent categories were retained in the model regardless of the statistical significance of individual category-level coefficients, with one category specified as the reference group. This approach ensured that categorical predictors were evaluated as complete variables and avoided selective exclusion of individual categories based solely on their category-specific *p*-values. The multivariable model accounted for potential confounding and estimated adjusted relationships between the predictors and MHC utilization; COR: crude odds ratio; AOR: Adjusted odds ration; 95%CI: ninety-five percent confidence interval.

After multivariable adjustment, female sex remained related to higher odds of MHC utilization (AOR = 1.95, 95% CI: 1.87–2.04; p < 0.001). Odds of MHC utilization decreased progressively with age compared with adults aged 40–49 years: 50–64 years (AOR = 0.80, 95% CI: 0.76–0.84; p < 0.001), 65–74 years (AOR = 0.65, 95% CI: 0.61–0.69; p < 0.001), and ≥75 years (AOR = 0.51, 95% CI: 0.47–0.54; p < 0.001). Non-Hispanic Whites had higher odds of MHC utilization (AOR = 1.70, 95% CI: 1.58–1.82; p < 0.001), whereas non-Hispanic Black (AOR = 0.81, 95% CI: 0.74–0.89; p < 0.001) and non-Hispanic Asian participants had lower odds (AOR = 0.54, 95% CI: 0.47–0.62; p < 0.001). Compared with ageing adults with less than a high school education, those with a high school diploma or GED, some college or an associate degree, and a bachelor’s degree or higher had 30%, 21%, and 12% lower odds of MHC utilization, respectively (AOR = 0.70, 95% CI: 0.64–0.76; p *<* 0.001; AOR = 0.79, 95% CI: 0.75–0.83; p *<* 0.001; and AOR = 0.88, 95% CI: 0.83–0.93; p *<* 0.001). Contrary to married ageing adults, participants who were neither married nor living with a partner had 23% higher odds of MHC utilization (AOR = 1.23, 95% CI: 1.18–1.28; p *<* 0.001). Relative to ageing adults residing in the Northeast, those in the Midwest had 10% higher odds of MHC utilization (AOR = 1.10, 95% CI: 1.03–1.17; p = 0.006). Comorbidity remained strongly related to MHC utilization, with AORs of 1.42 (95% CI: 1.35–1.49; p < 0.001) for one comorbidity and 2.23 (95% CI: 2.11–2.34; p < 0.001) for ≥2 comorbidities. Unlike ageing adults who did not smoke, those who smoked on some days and every day had 23% and 12% higher odds of MHC utilization, respectively (AOR = 1.23, 95% CI: 1.10–1.37; *p* < 0.001; and AOR = 1.12, 95% CI: 1.05–1.20; *p* = 0.001). Compared with adults of normal weight, those who were overweight and obese had 9% and 23% higher odds of MHC utilization, respectively (AOR = 1.09, 95% CI: 1.04–1.15; *p* < 0.001; and AOR = 1.23, 95% CI: 1.17–1.29; *p* < 0.001). Underweight adults had 15% lower odds of MHC utilization, although this association was borderline statistically significant (AOR = 0.85, 95% CI: 0.72–1.00; p = 0.050). Insurance coverage was also strongly related to MHC utilization (AOR = 2.51, 95% CI: 2.24–3.83; p < 0.001). Depression (AOR = 4.08, 95% CI: 3.83–4.34; p < 0.001) and anxiety (AOR = 3.49, 95% CI: 3.35–3.64; p < 0.001) remained the strongest independent correlates of MHC utilization (see Fig 3).

**Fig 3:**
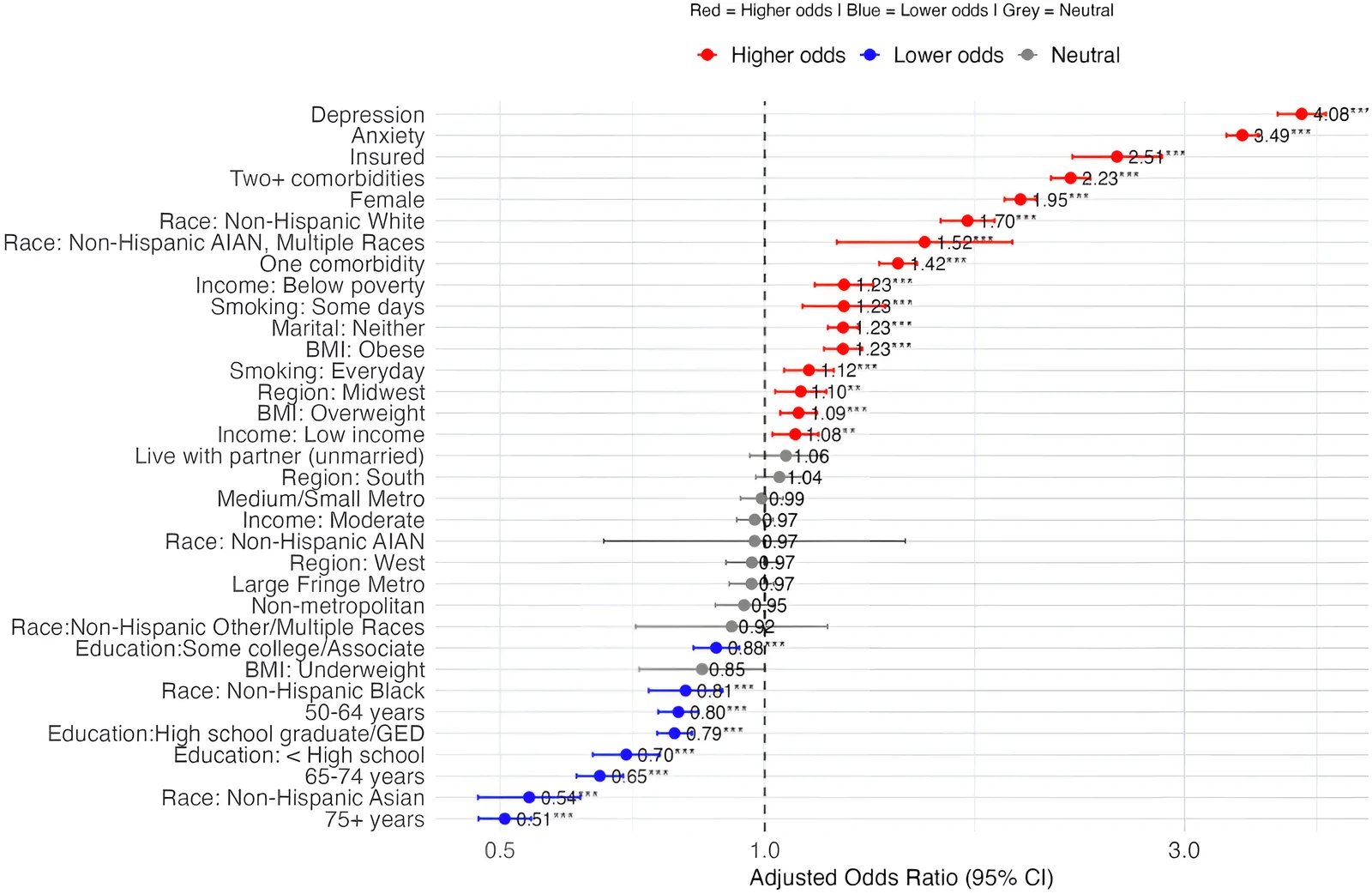
Primary model forest plot of factors associated with MHC utilization. The primary model includes depression and anxiety and presents the independent associations of demographic, socioeconomic, behavioral, and clinical characteristics with MHC utilization among middle-aged and older adults in the United States, based on the fully adjusted logistic regression model. The vertical dashed line at an odds ratio (OR) of 1.0 represents the null value, indicating no association with MHC utilization. *p* < 0.05; *p < 0.01; **p < 0.001.

The secondary model without depression and anxiety showed a similar pattern of direction with the primary model, with a slight variation in both the crude and adjusted odds (see S3 Table and S1 Fig).

## Discussion

Using nationally representative NHIS data from 2019–2024, this study examined MHC utilization and its related factors among U.S. middle-aged and older adults. Approximately one in five adults reported MHC use in the preceding 12 months (see Table 1 and S2 Table). Patterns of utilization varied across the predisposing, enabling, and need domains of Andersen’s Behavioral Model. Predisposing factors were related to notable differences in MHC utilization: females, non-Hispanic White adults, unmarried adults, and those who smoked occasionally or daily, as well as overweight and obese adults, reported greater MHC use, whereas adults aged 50–64, 65–74, and ≥75 years and non-Hispanic Black and Asian adults reported lower MHC utilization. Enabling factors also shaped utilization, with greater MHC use among adults with lower income, health insurance coverage, and residence in the Midwest, while higher educational attainment was related to lower MHC utilization. Need factors showed a consistent pattern, with MHC use increasing among adults with one or ≥2 comorbidities, depression, or anxiety (see Fig 3).

In this study, approximately one-fifth of ageing adults utilized MHC services (see Table 1) despite a substantial burden of mental health need (see Fig 4). This finding is consistent with prior evidence that MHC utilization remains low among older adults despite considerable unmet need [41–43]. Previous studies reported that only 6.5% of community-dwelling older adults received MHC in the preceding year, while 65.9% of those with major depressive disorder and 72.5% of those with anxiety did not receive care [41]. Similarly, fewer than 40% of older adults with serious psychological distress received MHC [42], and approximately 70% of older adults with mood or anxiety disorders reported no service use [44]. Although national MHC utilization increased from 7.0% in 1999 to 11.3% in 2018, disparities persisted across sociodemographic and health characteristics [25]. Our findings extend this evidence by showing that, despite increased recognition and availability of MHC over time, a substantial gap between mental health need and service utilization persists among ageing adults. This gap may reflect the combined influence of illness characteristics, perceived need [43,45], attitudes toward care, and sociodemographic barriers [41–43,45].

**Fig 4:**
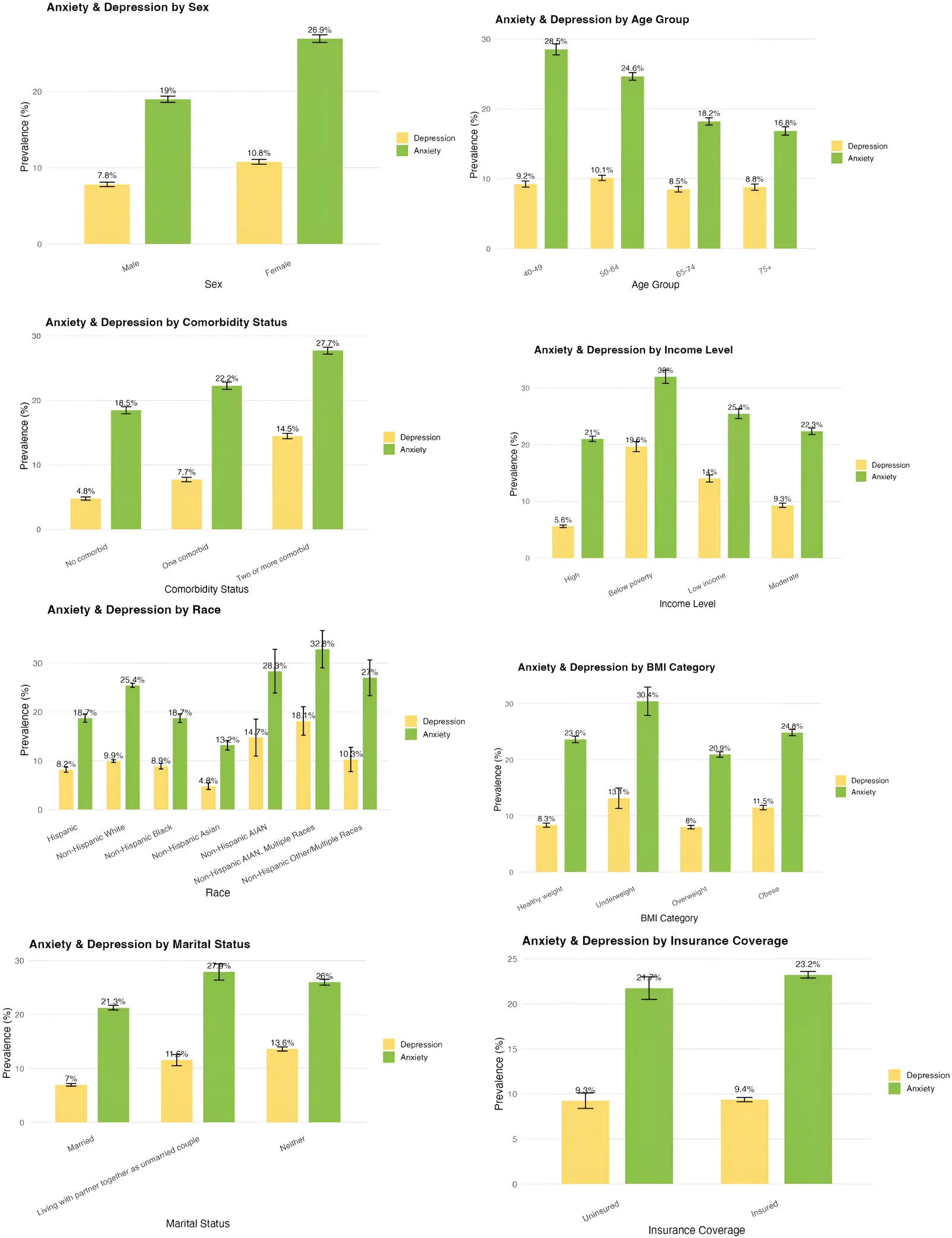
Distribution of anxiety and depression across participant characteristics. The figure provides a descriptive visual summary of anxiety and depression across participant characteristics, including sex, age group, comorbidity status, income level, race/ethnicity, BMI, insurance coverage, and marital status. These panels visually summarize patterns described in the manuscript and are intended to contextualize the primary findings rather than introduce new analyses or inferential results.

Females had approximately twice the odds of males to utilize MHC (see Fig 3). The results are consistent with global evidence that females generally take a more proactive approach to health care, including mental health support [46–48]. Multiple factors likely contribute to this disparity. Epidemiologically, females exhibit a higher prevalence of depressive and anxiety disorders, a pattern observed in this cohort (see Fig 4), which increases the likelihood of mental health service use (see Table 2). Similarly, females externally express their emotional distress and seek mental help, whereas males internalize these symptoms due to stigma and societal expectations [49], which may delay or prevent access to MHC services [50,51]. Unlike males, females’ frequent engagement with health care systems through reproductive and preventive services may create additional opportunities for mental health screening and referral [52].

Race was associated with MHC utilization. Compared with Hispanic adults, non-Hispanic White adults had higher odds of MHC utilization, whereas non-Hispanic Black and Asian adults had lower odds (see Fig 3). These findings are consistent with persistent racial and ethnic disparities in MHC utilization across U.S. populations [53]. Prior studies have reported lower treatment initiation and adequacy among Black and Latino older adults than among White adults, as well as lower outpatient mental health service use among African American adults and several other racial and ethnic minority groups [54]. More recent national evidence similarly shows higher MHC utilization among White adults than among Black, Hispanic, and Asian adults [55]. These disparities may partly reflect differences in structural access, treatment attitudes, stigma, and barriers to care [54,56–58]. For example, greater Latino isolation and centralization have been associated with residence in psychiatrist shortage areas, whereas greater African American concentration has been associated with greater neighborhood access to psychiatrists, suggesting that the availability of mental health providers may vary across racial and ethnic communities [58]. Differences in treatment attitudes and stigma may further contribute, including greater mental health stigma among Black adults and greater discomfort discussing mental health concerns among Hispanic and Asian adults [54,56,57].

Unmarried adults were more likely to utilize MHC services than married adults (see Fig 3). This association may reflect differences in social support, as marriage can provide emotional and practical resources that buffer stress and reduce reliance on formal MHC [59–62]. Conversely, cohabiting or living as an unmarried partner has been associated with poorer overall health and greater psychological distress [63], while reduced economic resources, less consistent social support, loneliness, and social isolation may further increase depressive symptoms and the need for professional mental health care [63–65]. Given the potential protective role of marriage in healthy aging, these findings underscore the value of strengthening social connectedness and implementing targeted outreach for socially isolated or unmarried adults to facilitate engagement with MHC services.

Contrary to non-smokers, ageing adults who smoked occasionally or daily were more likely to utilize MHC services (see Fig 3). Although tobacco smoking is less prevalent among older adults [66], smoking is associated with a higher risk of mental health disorders [30, 67, 68], including anxiety and depressive symptoms [69,70], which may indicate greater underlying mental health needs among older adults who smoke. These needs may increase contact with healthcare providers and the likelihood of referral to MHC during medical visits [30], thereby contributing to the higher MHC utilization observed among occasional and daily smokers [25].

Relative to ageing adults within the normal weight range, ageing adults who were overweight or obese showed a progressive increase in MHC utilization, whereas an inverse pattern was observed among those who were underweight; however, the latter association did not reach statistical significance (see Table 2). Higher BMI outside the normal range may reflect greater cardiometabolic and overall disease burden, as both overweight and obesity are associated with an increased risk of hypertension [71–73] and diabetes [72,74,75]. These chronic diseases may, in turn, increase vulnerability to mental health disorders [76,77] as shown in this cohort (see Fig 4), potentially contributing to greater demand for and utilization of MHC services.

Contrary to expectations [25,78,79], although MHC utilization increased with age and morbidity in the descriptive analysis (see Fig 1), multivariable regression showed progressively lower odds of MHC utilization with age (see Table 2), consistent with findings from the Health Care for Communities survey by Braden et al., which reported lower mental health service use among adults with chronic disease-related pain [80]. This discrepancy may reflect the increasing comorbidity burden with age alongside relatively stable depression and increasing anxiety (see Fig 4), as well as age-specific barriers such as mobility limitations, limited familiarity with MHC, generational stigma, and reliance on informal support [81,82]. These results underscore the need for age-tailored strategies, including workplace-based support and flexible care options for middle-aged adults [83–85]and targeted outreach, telehealth, community programs, and stigma reduction efforts for older adults [86–88].

Income is an important enabling factor that influences MHC utilization in the U.S. population [25,89]. In this study, aging adults with lower incomes and those below the federal poverty level had higher odds of MHC utilization than their higher-income counterparts (see Table 2). This finding is consistent with Wang and colleagues’ analysis of National Health and Nutrition Examination Survey (NHANES) data from 1999–2018, which found that a lower family poverty-income ratio was associated with higher odds of MHC utilization [25]. However, it contrasts with findings from Lee and colleagues’ analysis of the nationally representative 2020 Household Pulse Survey, which reported 50% higher odds of delayed mental health care among adults with incomes < $25,000 [89]. Similarly, Gao and Olfson, using 2018–2021 Medical Expenditure Panel Survey data, reported higher out-of-pocket MHC spending among individuals below the federal poverty line [90]. These differences may reflect variation in the outcome assessed, as MHC utilization does not necessarily indicate timely or adequate access to care. Despite higher utilization, our findings also showed a greater mental health burden among lower-income adults (see Fig 4), consistent with evidence that financial hardship may undermine cognitive resilience [91].

Being insured was associated with nearly three-fold higher odds of MHC utilization compared with being uninsured (see Table 2). This finding is consistent with evidence from the National Survey on Drug Use and Health, which showed that enrollment in public insurance programs, including Medicare, Medicaid, and Department of Veterans Affairs (VA)/military insurance, was associated with greater odds of receiving MHC [5, 92,93]. Similarly, Koroma et al., using the 2023 NHIS, reported higher odds of MHC utilization among insured adults [55]. Although our analysis pooled six NHIS waves, the consistency of these findings supports insurance coverage as an important enabling factor for MHC access [55]. However, the substantial missingness and consequently limited complete cases for insurance subtype data precluded the assessment of whether MHC utilization differed by specific insurance type. Yet, subsidization of insurance coverage to reduce out-of-pocket spending [94] will be critically essential to improve MHC use [5,93,95,96].

Residence in the Midwest was associated with a 10% higher likelihood of MHC utilization (see Table 2). This observation is consistent with evidence that place-specific factors account for substantial variation in mental health service use among older adults [97]. McConnell et al. similarly reported higher rates of mental health-related emergency department visits in Ohio, a Midwestern state, than in Nevada and Arizona, in the Western and Southwestern regions, respectively [98].. However, our findings contrast with earlier results from the Collaborative Psychiatric Epidemiology Surveys, which identified greater unmet MHC needs among Black and Latino adults in the Midwest [26]. These differences point to changes in service availability and access over time, although the cross-sectional design precludes causal inference. Continued efforts to expand accessible and equitable MHC services across regions are warranted.

Education level is a pertinent predictor of MHC utilization [25]. We observed a progressive decrease in the likelihood of MHC utilization with increasing educational attainment (see Table 2 and Fig 3). This finding is consistent with evidence from the Health and Retirement Study, an ongoing longitudinal study of adults aged ≥50 years, which showed that higher educational attainment was associated with fewer depressive symptoms over 24 years [99]. Similarly, data from the Medical Expenditure Panel Survey indicated that adults with less than a high school education were less likely to report good mental health [100]. These findings suggest that the lower MHC utilization observed among more highly educated adults may partly reflect a lower burden of mental health symptoms rather than reduced access to care. Given evidence from *The Lancet* 2024 reports that mental health distress increases dementia risk, while higher educational attainment confers cognitive resilience [101], these results suggest early identification and targeted MHC outreach among adults with lower educational attainment, who may experience a greater burden of mental health needs.

Compared with those without comorbidities, MHC utilization showed a gradient increase among participants with one and ≥2 comorbidities (see Table 2). This finding is consistent with a retrospective cohort study of Medicare-insured older adults, in which Prajapati and colleagues reported greater annual inpatient, outpatient, and emergency department use among beneficiaries with cardiovascular disease but no mental health disorders than among those with neither condition [102]. Greater chronic disease burden may increase treatment burden and psychological stress, thereby increasing the need for MHC [103–105]. In this study, participants with one comorbidity had 42% higher odds of MHC utilization, whereas those with ≥2 comorbidities had 2.23 times increase inodds (123% higher), indicating a graded relationship (see Fig 2). However, the relatively low overall utilization suggests that increased need may not translate into care because of barriers such as cost, stigma, limited awareness, and fragmented services. These findings necessitate the adoption of integrated care models that address the mental and physical health needs of adults with multiple chronic conditions [47,106–109].

In this cohort, depression and anxiety were prevalent and varied across sociodemographic and socioeconomic characteristics (see Fig 4). The presence of these conditions does not necessarily translate into MHC utilization [41,44,110], as many older adults may internalize mental health difficulties because of stigma and consequently not seek care [56]. However, these patterns are consistent with findings by Adepoju et al., who reported marginally higher receipt of office-based MHC and mental health-related emergency department visits among older adults with mental health difficulties [111], and Blais et al., who found higher odds of MHC utilization among adults with depression and psychiatric symptoms in the National Health and Resilience in Veterans Study [112]. Further, the results from this study broadly align with the well-established association between psychiatric symptom severity and MHC-seeking behavior [93,113,114]. These results underscore the importance of integrating routine MHC screening into primary care and chronic disease management to facilitate early detection and timely treatment, improve outcomes, and reduce the burden of untreated psychiatric disorders.

This study has several strengths. The large, nationally representative NHIS sample provides substantial statistical power and supports the generalizability of the findings to U.S. adults aged 40 years and older. Pooling six consecutive survey years improves the precision and stability of estimates while reducing the influence of year-specific sampling variation. The use of survey-weighted multivariable logistic regression accounts for the complex NHIS sampling design and permits adjustment for a broad range of prespecified covariates. The analysis also examines MHC utilization across multiple domains of the Andersen Behavioral Model**,** incorporating predisposing, enabling, and need-related factors, with comorbidity burden examined as a key clinical factor among middle-aged and older adults. Standardized NHIS measures further facilitate consistent assessment across survey waves. In addition, the population-based design provides findings relevant to clinical practice and public health planning, particularly in identifying groups that may experience lower MHC utilization. Finally, the study was reported in accordance with the Strengthening the Reporting of Observational Studies in Epidemiology (STROBE) guidelines for cross-sectional studies, with the completed STROBE checklist provided as supplementary material (see S1 Checklist). Together, these features strengthen the methodological transparency, population relevance, and interpretability of the findings. Nonetheless, several limitations should be considered. The cross-sectional design limits causal inference and prevents determination of temporality between predictors and MHC utilization, while reliance on self-reported measures may introduce recall or social desirability bias. Restriction of comorbidity to six conditions may have underestimated the overall burden of chronic disease by excluding other conditions captured by the NHIS. Insurance type and coverage were not examined in detail, limiting assessment of differences across specific coverage arrangements. Moreover, the overall measure of MHC utilization did not distinguish among counseling or psychotherapy, prescription medication for mental health conditions, and visits to mental health specialists, limiting assessment of service-specific utilization patterns. Although age and comorbidity trends were examined, survey year was not included as a covariate in the primary models or used for formal temporal trend assessment. Changes in NHIS survey design, including the 2019 redesign and changes in data collection during 2020, as well as reduced response rates and the rapid expansion of telehealth during 2020–2021, may have affected comparability across pooled survey waves. Accordingly, patterns across the 2019–2024 period should be interpreted cautiously and should not be attributed solely to changes in MHC utilization. The public-use NHIS also provides limited geographic detail beyond Census region, precluding assessment of important supply-side factors such as local provider availability, provider density, network adequacy, and appointment availability. Thus, a non-significant adjusted relationship between non-metropolitan residence and MHC utilization should not be interpreted as evidence that geographic or local access barriers are unimportant. Finally, unmeasured factors, including cultural beliefs and health system characteristics, may contribute to differences in MHC utilization.

## Conclusion

In summary, approximately one in five U.S. adults aged 40 years and older reported MHC use in the preceding 12 months. MHC utilization varied across predisposing, enabling, and need-related factors. Utilization was higher among females, non-Hispanic White adults, unmarried adults, smokers, and adults with overweight or obesity, whereas it was lower across the older age groups (50–64, 65–74, and ≥75 years) and among non-Hispanic Black and Asian adults, relative to their respective reference groups. Among enabling factors, utilization was higher among insured adults and those with lower income, while higher educational attainment was associated with lower utilization. Need-related factors showed consistent relationships with utilization, with higher use among adults with one or ≥2 comorbidities and among those with depression or anxiety. These findings indicate that MHC utilization is related to multiple demographic, socioeconomic, behavioral, and clinical factors among adults aged 40 years and older. The findings also highlight populations that may experience lower utilization despite greater potential need. Given the cross-sectional design, these relationships should not be interpreted as causal. Efforts to improve equitable access to mental health care should consider differences in insurance coverage, socioeconomic circumstances, comorbidity burden, and mental health needs, while recognizing that the specific mechanisms underlying these relationships require further investigation. Future research should examine how these relationships vary across specific types of mental health services and care settings.

## Supporting information

S1 Table Collinearity diagnostics for predictors. (DOCX)

S2 Table Description of the weighted sample MHC utilization among middle-aged and older adults in the U.S. (DOCX)

S3 Table Univariate and multiple logistic regression of factors associated with MHC utilization, excluding depression and anxiety. (DOCX)

S1 Checklist STROBE Statement—checklist of items that should be included in reports of observational studies. (DOCX)

S1 Fig Secondary model forest plot of factors associated with MHC utilization. The secondary model excludes depression and anxiety and presents the independent associations of demographic, socioeconomic, behavioral, and clinical characteristics with MHC utilization among middle-aged and older adults in the United States, based on the fully adjusted logistic regression model. The vertical dashed line at an odds ratio (OR) of 1.0 represents the null value, indicating no association with MHC utilization. *p* < 0.05; *p < 0.01; **p < 0.001.
(TIF)

## Abbreviations

AOR: Adjusted Odds Ratio
BMI: Body Mass Index
CI: Confidence Interval
COR: Crude Odds Ratio
FPL: Federal Poverty Level
HHS: U.S. Department of Health and Human Services
MHC: Mental Health Care
NHANES: National Health and Nutrition Examination Survey
NHIS: National Health Interview Survey
OR: Odds Ratio
SE: Standard Error
SES: Socioeconomic Status
U.S: United States

## References

[1] Reynolds K, Pietrzak RH, El-Gabalawy R, Mackenzie CS, Sareen J. Prevalence of psychiatric disorders in U.S. older adults: Findings from a nationally representative survey. World Psychiatry. 2015;14(1):74–81. doi: 10.1002/wps.20193 25655161 PMC4329900

[2] Reinert M, Fritze D, Nguyen T. The state of mental health in America 2024. 2024.

[3] Zhao G, Ford ES, Dhingra S, Li C, Strine TW, Mokdad AH. Depression and anxiety among US adults: Associations with body mass index. Int J Obes (Lond). 2009;33(2):257–66. doi: 10.1038/ijo.2008.268 19125163

[4] Sheehan A, Walsh R, Liu R. Racial and ethnic trends in mental health service utilisation and perceived unmet need in the USA. J Epidemiol Community Health. 2024;78(4):228–34. doi: 10.1136/jech-2023-220683 38242681 PMC10988996

[5] Walker ER, Cummings JR, Hockenberry JM, Druss BG. Insurance status, use of mental health services, and unmet need for mental health care in the United States. Psychiatr Serv. 2015;66(6):578–84. doi: 10.1176/appi.ps.201400248 25726980 PMC4461054

[6] Wang PS, Lane M, Olfson M, Pincus HA, Wells KB, Kessler RC. Twelve-month use of mental health services in the United States: Results from the National Comorbidity Survey Replication. Arch Gen Psychiatry. 2005;62(6):629–40. doi: 10.1001/archpsyc.62.6.629 15939840

[7] Karlin BE, Duffy M, Gleaves DH. Patterns and predictors of mental health service use and mental illness among older and younger adults in the United States. Psychological Services. 2008;5(3):275–94. doi: 10.1037/1541-1559.5.3.275

[8] Lin EHB, Von Korff M, Alonso J, Angermeyer MC, Anthony J, Bromet E, et al. Mental disorders among persons with diabetes--results from the World Mental Health Surveys. J Psychosom Res. 2008;65(6):571–80. doi: 10.1016/j.jpsychores.2008.06.007 19027447 PMC3672403

[9] Kretchy IA, Owusu-Daaku FT, Danquah SA. Mental health in hypertension: Assessing symptoms of anxiety, depression and stress on anti-hypertensive medication adherence. Int J Ment Health Syst. 2014;8:25. doi: 10.1186/1752-4458-8-25 24987456 PMC4077111

[10] Osei Duah Junior I, Asare WO, Johnson EJ, Boateng C, Acheampong HO, Ben Kumah D, et al. An integrative examination of psychological distress and its nutritional and visual correlates among young adults in Ghana. PLoS One. 2025;20(11):e0329869. doi: 10.1371/journal.pone.0329869 41248210 PMC12622780

[11] Scott J, Scott EM, Hermens DF, Naismith SL, Guastella AJ, White D, et al. Functional impairment in adolescents and young adults with emerging mood disorders. Br J Psychiatry. 2014;205(5):362–8. doi: 10.1192/bjp.bp.113.134262 25213156

[12] Rosa AR, Reinares M, Michalak EE, Bonnin CM, Sole B, Franco C, et al. Functional impairment and disability across mood states in bipolar disorder. Value Health. 2010;13(8):984–8. doi: 10.1111/j.1524-4733.2010.00768.x 20667057

[13] Saris IMJ, Aghajani M, van der Werff SJA, van der Wee NJA, Penninx BWJH. Social functioning in patients with depressive and anxiety disorders. Acta Psychiatr Scand. 2017;136(4):352–61. doi: 10.1111/acps.12774 28767127 PMC5601295

[14] Ducat L, Philipson LH, Anderson BJ. The mental health comorbidities of diabetes. JAMA. 2014;312(7):691–2. doi: 10.1001/jama.2014.8040 25010529 PMC4439400

[15] Hutter N, Schnurr A, Baumeister H. Healthcare costs in patients with diabetes mellitus and comorbid mental disorders--A systematic review. Diabetologia. 2010;53(12):2470–9. doi: 10.1007/s00125-010-1873-y 20700575

[16] Angst F, Stassen HH, Clayton PJ, Angst J. Mortality of patients with mood disorders: Follow-up over 34-38 years. J Affect Disord. 2002;68(2–3):167–81. doi: 10.1016/s0165-0327(01)00377-9 12063145

[17] Chen C, Chan H-Y, Yeh L-L, Pan Y-J. Longitudinal factors associated with mortality in older patients with mood disorders. J Affect Disord. 2021;278:12500. doi: 10.1016/j.jad.2020.09.097 33035948

[18] Kessler RC, Merikangas KR, Wang PS. Prevalence, comorbidity, and service utilization for mood disorders in the United States at the beginning of the twenty-first century. Annu Rev Clin Psychol. 2007;3:137–58. doi: 10.1146/annurev.clinpsy.3.022806.091444 17716051

[19] Elhai JD, Ford JD. Correlates of mental health service use intensity in the National Comorbidity Survey and National Comorbidity Survey Replication. Psychiatr Serv. 2007;58(8):1108–15. doi: 10.1176/ps.2007.58.8.1108 17664523

[20] Babitsch B, Gohl D, von Lengerke T. Re-revisiting Andersen’s behavioral model of health services use: A systematic review of studies from 1998-2011. Psychosoc Med. 2012;9:Doc11. doi: 10.3205/psm000089 23133505 PMC3488807

[21] Andersen RM. Revisiting the behavioral model and access to medical care: Does it matter?. J Health Soc Behav. 1995;36(1):1–10. doi: 10.2307/2137284 7738325

[22] Andersen R, Newman JF. Societal and individual determinants of medical care utilization in the United States. Milbank Mem Fund Q Health Soc. 1973;51(1):95–124. doi: 10.2307/3349613 4198894

[23] Aday LA, Andersen R. A framework for the study of access to medical care. Health Serv Res. 1974;9(3):208–20. 4436074 PMC1071804

[24] Andersen RM. National health surveys and the behavioral model of health services use. Med Care. 2008;46(7):647–53. doi: 10.1097/MLR.0b013e31817a835d 18580382

[25] Wang J, Qiu Y, Zhu X. Trends of mental health care utilization among US adults from 1999 to 2018. BMC Psychiatry. 2023;23(1):665. doi: 10.1186/s12888-023-05156-2 37700243 PMC10496400

[26] Kim G, Dautovich N, Ford K-L, Jimenez DE, Cook B, Allman RM, et al. Geographic variation in mental health care disparities among racially/ethnically diverse adults with psychiatric disorders. Soc Psychiatry Psychiatr Epidemiol. 2017;52(8):939–48. doi: 10.1007/s00127-017-1401-1 28589236

[27] Roberts T, Miguel Esponda G, Krupchanka D, Shidhaye R, Patel V, Rathod S. Factors associated with health service utilisation for common mental disorders: A systematic review. BMC Psychiatry. 2018;18(1):262. doi: 10.1186/s12888-018-1837-1 30134869 PMC6104009

[28] Harris KM, Edlund MJ, Larson S. Racial and ethnic differences in the mental health problems and use of mental health care. Med Care. 2005;43(8):775–84. doi: 10.1097/01.mlr.0000170405.66264.23 16034291

[29] Dobalian A, Rivers PA. Racial and ethnic disparities in the use of mental health services. J Behav Health Serv Res. 2008;35(2):128–41. doi: 10.1007/s11414-007-9097-8 18074230

[30] Cook BL, Wayne GF, Kafali EN, Liu Z, Shu C, Flores M. Trends in smoking among adults with mental illness and association between mental health treatment and smoking cessation. JAMA. 2014;311(2):172–82. doi: 10.1001/jama.2013.284985 24399556 PMC5555156

[31] Shen C, Sambamoorthi U, Rust G. Co-occurring mental illness and health care utilization and expenditures in adults with obesity and chronic physical illness. Dis Manag. 2008;11(3):153–60. doi: 10.1089/dis.2007.0012 18564027

[32] Weissman JD, Russell D, Ansah P, Jay M. Disparities in healthcare utilization among adults with obesity in the United States, findings from the NHIS: 2006–2015. Popul Res Policy Rev. 2019;38(3):403–15. doi: 10.1007/s11113-018-09507-w

[33] Li C, Ford ES, Strine TW, Mokdad AH. Prevalence of depression among U.S. adults with diabetes: Findings from the 2006 behavioral risk factor surveillance system. Diabetes Care. 2008;31(1):105–7. doi: 10.2337/dc07-1154 17934145

[34] Wilk AS, Hu J-C, Chehal P, Yarbrough CR, Ji X, Cummings JR. National estimates of mental health needs among adults with self-reported CKD in the United States. Kidney Int Rep. 2022;7(7):1630–42. doi: 10.1016/j.ekir.2022.04.088 35812303 PMC9263246

[35] Rozario SS, Masho SW. The associations between mental health status, hypertension, and hospital inpatient visits in women in the United States. Am J Hypertens. 2018;31(7):804–10. doi: 10.1093/ajh/hpy065 29897396

[36] Weissman JD, Russell D, Ansah P, Jay M. Disparities in healthcare utilization among adults with obesity in the United States, findings from the NHIS: 2006–2015. Popul Res Policy Rev. 2019;38(3):403–15. doi: 10.1007/s11113-018-09507-w

[37] Lilienthal K, Possemato K, Funderburk J, Wade M, Eaker A, Beehler GP. Predisposing characteristics, enabling factors, and need as predictors of integrated behavioral health utilization. J Behav Health Serv Res. 2017;44(2):263–73. doi: 10.1007/s11414-016-9496-9 26780207

[38] Yang JC, Roman-Urrestarazu A, McKee M, Brayne C. Demographic, socioeconomic, and health correlates of unmet need for mental health treatment in the United States, 2002–16: evidence from the national surveys on drug use and health. International journal for equity in health. 2019;18(1):122. 10.1186/s12939-019-1026-y PMC6683484 31382979

[39] Mojtabai R, Jorm AF. Trends in psychological distress, depressive episodes and mental health treatment-seeking in the United States: 2001-2012. J Affect Disord. 2015;174:556–61. doi: 10.1016/j.jad.2014.12.039 25556674

[40] Rodriguez-Seijas C, Eaton NR, Stohl M, Mauro PM, Hasin DS. Mental disorder comorbidity and treatment utilization. Compr Psychiatry. 2017;79:89–97. doi: 10.1016/j.comppsych.2017.02.003 28215792 PMC5550376

[41] Garrido MM, Kane RL, Kaas M, Kane RA. Use of mental health care by community-dwelling older adults. J Am Geriatr Soc. 2011;59(1):50–6. doi: 10.1111/j.1532-5415.2010.03220.x 21198461 PMC3050003

[42] Han B, Gfroerer JC, Colpe LJ, Barker PR, Colliver JD. Serious psychological distress and mental health service use among community-dwelling older U.S. adults. Psychiatric Services. 2011;62:291–8. 10.1176/ps.62.3.pss6203_0291 21363901

[43] Klap R, Unroe KT, Unützer J. Caring for mental illness in the United States: A focus on older adults. Am J Geriatr Psychiatry. 2003;11(5):517–24. doi: 10.1097/00019442-200309000-00006 14506085

[44] Byers AL, Arean PA, Yaffe K. Low use of mental health services among older Americans with mood and anxiety disorders. Psychiatric Services. 2012;63:66–72. 10.1176/appi.ps.201100121 PMC3726310 22227762

[45] Mackenzie CS, Pagura J, Sareen J. Correlates of perceived need for and use of mental health services by older adults in the collaborative psychiatric epidemiology surveys. Am J Geriatr Psychiatry. 2010;18(12):1103–15. doi: 10.1097/JGP.0b013e3181dd1c06 20808105 PMC2992082

[46] Nishi D, Susukida R, Usuda K, Mojtabai R, Yamanouchi Y. Trends in the prevalence of psychological distress and the use of mental health services from 2007 to 2016 in Japan. J Affect Disord. 2018;239:208–13. doi: 10.1016/j.jad.2018.07.016 30025309

[47] Olfson M, Wang S, Wall M, Marcus SC, Blanco C. Trends in serious psychological distress and outpatient mental health care of US adults. JAMA Psychiatry. 2019;76(2):152–61. doi: 10.1001/jamapsychiatry.2018.3550 30484838 PMC6439744

[48] Neighbors HW, Caldwell C, Williams DR, Nesse R, Taylor RJ, Bullard KM, et al. Race, ethnicity, and the use of services for mental disorders: Results from the National Survey of American Life. Arch Gen Psychiatry. 2007;64(4):485–94. doi: 10.1001/archpsyc.64.4.485 17404125

[49] Möller-Leimkühler AM. Barriers to help-seeking by men: A review of sociocultural and clinical literature with particular reference to depression. J Affect Disord. 2002;71(1–3):1–9. doi: 10.1016/s0165-0327(01)00379-2 12167495

[50] Clark LH, Hudson JL, Haider T. Anxiety specific mental health stigma and help-seeking in adolescent males. J Child Fam Stud. 2020;29(7):1970–81. doi: 10.1007/s10826-019-01686-0

[51] Whitley R. Why do men have low rates of formal mental health service utilization? An analysis of social and systemic barriers to care and discussion of promising male-friendly practices. Men’s Issues and Men’s Mental Health: An Introductory Primer. Springer. 2021. p. 127–49.

[52] Hall KS, Harris LH, Dalton VK. Women’s preferred sources for primary and mental health care: Implications for reproductive health providers. Womens Health Issues. 2017;27(2):196–205. doi: 10.1016/j.whi.2016.09.014 27825589 PMC5357444

[53] Kim G, Parton JM, DeCoster J, Bryant AN, Ford KL, Parmelee PA. Regional variation of racial disparities in mental health service use among older adults. Gerontologist. 2013;53(4):618–26. doi: 10.1093/geront/gns107 22859437 PMC3709841

[54] Jimenez DE, Cook B, Bartels SJ, Alegría M. Disparities in mental health service use of racial and ethnic minority elderly adults. J Am Geriatr Soc. 2013;61(1):18–25. doi: 10.1111/jgs.12063 23252464 PMC3545089

[55] Koroma MI, Inungu JN, Adu-Serwaah M, Sultana S, Younis MZ, Iheduru-Anderson K. Disparities in adult mental health service utilization in the United States: A cross-sectional study. ELECTRON J GEN MED. 2025;22(5):em683. doi: 10.29333/ejgm/16747

[56] Conner KO, Copeland VC, Grote NK, Koeske G, Rosen D, Reynolds CF, et al. Mental health treatment seeking among older adults with depression: The impact of stigma and race. Am J Geriatr Psychiatry. 2010;18(6):531–43. doi: 10.1097/JGP.0b013e3181cc0366 20220602 PMC2875324

[57] Sorkin DH, Murphy M, Nguyen H, Biegler KA. Barriers to mental health care for an ethnically and racially diverse sample of older adults. J Am Geriatr Soc. 2016;64(10):2138–43. doi: 10.1111/jgs.14420 27565017 PMC5937991

[58] Dinwiddie GY, Gaskin DJ, Chan KS, Norrington J, McCleary R. Residential segregation, geographic proximity and type of services used: Evidence for racial/ethnic disparities in mental health. Soc Sci Med. 2013;80:67–75. doi: 10.1016/j.socscimed.2012.11.024 23312305 PMC4119020

[59] Cutrona CE: Social support in couples: Marriage as a resource in times of stress: Sage Publications; 1996.

[60] Thoits PA. Mechanisms linking social ties and support to physical and mental health. J Health Soc Behav. 2011;52(2):145–61. doi: 10.1177/0022146510395592 21673143

[61] Wong JS, Waite LJ. Marriage, social networks, and health at older ages. Journal of Population Ageing. 2015;8(1):7–25. 10.1007/s12062-014-9110-y PMC4531842 26279707

[62] Wickrama KAS, King VA, O’Neal CW, Lorenz FO. Stressful work trajectories and depressive symptoms in middle-aged couples: Moderating effect of marital warmth. J Aging Health. 2017:898264317736135. doi: 10.1177/0898264317736135 29254406 PMC8340926

[63] Lamidi EO, Nash SP. Two decades of change in living arrangements and health of middle-aged and older adults in the U.S., 1997-2018. J Appl Gerontol. 2022;41(5):1407–20. doi: 10.1177/07334648221074685 35209757

[64] Hewitt B, Turrell G, Giskes K. Marital loss, mental health and the role of perceived social support: Findings from six waves of an Australian population based panel study. J Epidemiol Community Health. 2012;66(4):308–14. doi: 10.1136/jech.2009.104893 20966446

[65] Brown SL, Bulanda JR, Lee GR. The significance of nonmarital cohabitation: Marital status and mental health benefits among middle-aged and older adults. J Gerontol B Psychol Sci Soc Sci. 2005;60(1):S21-9. doi: 10.1093/geronb/60.1.s21 15643043

[66] Blazer DG, Wu L-T. Patterns of tobacco use and tobacco-related psychiatric morbidity and substance use among middle-aged and older adults in the United States. Aging Ment Health. 2012;16(3):296–304. doi: 10.1080/13607863.2011.615739 22292514 PMC3323715

[67] Lawrence D, Mitrou F, Zubrick SR. Smoking and mental illness: Results from population surveys in Australia and the United States. BMC Public Health. 2009;9:285. doi: 10.1186/1471-2458-9-285 19664203 PMC2734850

[68] McClave AK, McKnight-Eily LR, Davis SP, Dube SR. Smoking characteristics of adults with selected lifetime mental illnesses: Results from the 2007 National Health Interview Survey. Am J Public Health. 2010;100(12):2464–72. doi: 10.2105/AJPH.2009.188136 20966369 PMC2978196

[69] Morrell HER, Cohen LM. Cigarette smoking, anxiety, and depression. J Psychopathol Behav Assess. 2006;28(4):281–95. doi: 10.1007/s10862-005-9011-8

[70] Smith PH, Mazure CM, McKee SA. Smoking and mental illness in the U.S. population. Tob Control. 2014;23(e2):e147-53. doi: 10.1136/tobaccocontrol-2013-051466 24727731 PMC4201650

[71] Wang J, Yang Q-Y, Chai D-J, Su Y, Jin Q-Z, Wang J-H. The relationship between obesity associated weight-adjusted waist index and the prevalence of hypertension in US adults aged ≥60 years: A brief report. Front Public Health. 2023;11:1210669. doi: 10.3389/fpubh.2023.1210669 37869197 PMC10587597

[72] Chen AS, Chetty AK, Batsis JA, Lipska KJ, Hajduk AM. Conditions attributable to obesity in older adults in the U.S.: A cross-sectional study. American Journal of Preventive Medicine. 2026;71(4):108419. 10.1016/j.amepre.2026.108419 PMC13235660 42150623

[73] Tu J, Chen H, Zeng Q, Chen L, Guo Y, Chen K. Trends in obesity prevalence among adults with hypertension in the United States, 2001 to 2023. Hypertension. 2025;82(3):498–508. doi: 10.1161/HYPERTENSIONAHA.124.24123 39758027

[74] Nguyen NT, Nguyen X-MT, Lane J, Wang P. Relationship between obesity and diabetes in a US adult population: Findings from the National Health and Nutrition Examination Survey, 1999-2006. Obes Surg. 2011;21(3):351–5. doi: 10.1007/s11695-010-0335-4 21128002 PMC3040808

[75] Chen Y, Lundeen EA, Koyama AK, Kompaniyets L, Andes LJ, Benoit SR, et al. Prevalence of testing for diabetes among US adults with overweight or obesity, 2016-2019. Prev Chronic Dis. 2023;20:E116. doi: 10.5888/pcd20.230173 38154119 PMC10756652

[76] Zhou J, Sherrill CH, Hwang AY, Lee S. Association between mental health and self-care behavior among older adults with diabetes according to Behavioral Risk Factor Surveillance System 2019. Prim Care Diabetes. 2023;17(3):250–4. doi: 10.1016/j.pcd.2023.02.005 36898951

[77] Maatouk I, Herzog W, Böhlen F, Quinzler R, Löwe B, Saum K-U, et al. Association of hypertension with depression and generalized anxiety symptoms in a large population-based sample of older adults. J Hypertens. 2016;34(9):1711–20. doi: 10.1097/HJH.0000000000001006 27341438

[78] Karlin BE, Duffy M, Gleaves DH. Patterns and predictors of mental health service use and mental illness among older and younger adults in the United States. Psychological Services. 2008;5(3):275–94. doi: 10.1037/1541-1559.5.3.275

[79] Neighbors HW, Woodward AT, Bullard KM, Ford BC, Taylor RJ, Jackson JS. Mental health service use among older African Americans: The National Survey of American Life. Am J Geriatr Psychiatry. 2008;16(12):948–56. doi: 10.1097/JGP.0b013e318187ddd3 19038893 PMC4846982

[80] Braden JB, Zhang L, Fan M-Y, Unützer J, Edlund MJ, Sullivan MD. Mental health service use by older adults: The role of chronic pain. Am J Geriatr Psychiatry. 2008;16(2):156–67. doi: 10.1097/JGP.0b013e31815a3ea9 18192496 PMC2749707

[81] Lavingia R, Jones K, Asghar-Ali AA. A Systematic Review of Barriers Faced by Older Adults in Seeking and Accessing Mental Health Care. J Psychiatr Pract. 2020;26(5):367–82. doi: 10.1097/PRA.0000000000000491 32936584

[82] Brown J, Evans-Lacko S, Aschan L, Henderson MJ, Hatch SL, Hotopf M. Seeking informal and formal help for mental health problems in the community: A secondary analysis from a psychiatric morbidity survey in South London. BMC Psychiatry. 2014;14(1):275. doi: 10.1186/s12888-014-0275-y 25292287 PMC4195997

[83] Li L, Lee Y, Lai DWL. Mental health of employed family caregivers in Canada: A gender-based analysis on the role of workplace support. Int J Aging Hum Dev. 2022;95(4):470–92. doi: 10.1177/00914150221077948 35124984 PMC9554159

[84] Pomaki G, Franche R-L, Murray E, Khushrushahi N, Lampinen TM. Workplace-based work disability prevention interventions for workers with common mental health conditions: A review of the literature. J Occup Rehabil. 2012;22(2):182–95. doi: 10.1007/s10926-011-9338-9 22038297

[85] Hogg B, Moreno-Alcázar A, Tóth MD, Serbanescu I, Aust B, Leduc C, Paterson C, Tsantilla F, Abdulla K, Cerga-Pashoja A: Supporting employees with mental illness and reducing mental illness-related stigma in the workplace: An expert survey. European archives of psychiatry and clinical neuroscience 2023;273(3):739–53. 10.1007/s00406-022-01443-3 PMC9305029 35867155

[86] Van Citters AD, Bartels SJ. A systematic review of the effectiveness of community-based mental health outreach services for older adults. Psychiatr Serv. 2004;55(11):1237–49. doi: 10.1176/appi.ps.55.11.1237 15534012

[87] Harerimana B, Forchuk C, O’Regan T. The use of technology for mental healthcare delivery among older adults with depressive symptoms: A systematic literature review. Int J Ment Health Nurs. 2019;28(3):657–70. doi: 10.1111/inm.12571 30666762

[88] Conner KO, McKinnon SA, Roker R, Ward CJ, Brown C. Mitigating the stigma of mental illness among older adults living with depression: The benefit of contact with a peer educator. Stigma and Health. 2018;3(2):93–101. doi: 10.1037/sah0000076

[89] Lee H, Singh GK. Monthly trends in access to care and mental health services by household income level during the COVID-19 pandemic, United States, April: December 2020. Health Equity. 2021;5(1):heq.2021.0036. 10.1089/heq.2021.0036 PMC8665787 34909547

[90] Gao YN, Olfson M. High out-of-pocket cost burden of mental health care for adult outpatients in the United States. Psychiatr Serv. 2025;76(2):200–3. doi: 10.1176/appi.ps.20240136 39257310 PMC11786981

[91] Iskandar M, Martindale J, Bynum JPW, Davis MA. Association between Family household income and cognitive resilience among older US adults: A cross-sectional study. J Prev Alzheimers Dis. 2024;11(5):1406–9. doi: 10.14283/jpad.2024.97 39350387 PMC12598677

[92] Choi NG, DiNitto DM, Marti CN. Relationship between the types of insurance coverage and outpatient mental health treatment use among older adults. J Appl Gerontol. 2016;35(12):1343–62. doi: 10.1177/0733464815577143 25800462

[93] Wang N, Xie X. Associations of health insurance coverage, mental health problems, and drug use with mental health service use in US adults: an analysis of 2013 National Survey on Drug Use and Health. Aging Ment Health. 2019;23(4):439–46. doi: 10.1080/13607863.2018.1441262 29469591

[94] Hattab Z, Doherty E, Ryan AM, O’Neill S. Heterogeneity within the Oregon Health Insurance Experiment: An application of causal forests. PLoS One. 2024;19(1):e0297205. doi: 10.1371/journal.pone.0297205 38236917 PMC10796043

[95] Carter SP, Cowan T, Snow A, Cerel J, Tucker R. Health insurance and mental health care utilization among adults who identify as transgender and gender diverse. Psychiatr Serv. 2020;71(2):151–7. doi: 10.1176/appi.ps.201900289 31658897

[96] Tzenios N. The determinants of access to healthcare: A review of individual, structural, and systemic factors. Journal of Humanities and Applied Science Research. 2019;2(1):1–14.

[97] Ding H. Geographic Variation in mental health treatment utilization: Evidence from migration. American Economic Journal: Economic Policy. 2026;18(1):35–68. doi: 10.1257/pol.20230402

[98] McConnell KJ, Watson K, Choo E, Zhu JM. Geographical variations in emergency department visits for mental health conditions for medicaid beneficiaries. Health Aff (Millwood). 2023;42(2):172–81. doi: 10.1377/hlthaff.2022.00796 36745838 PMC11203219

[99] Assari S. Combined effects of ethnicity and education on burden of depressive symptoms over 24 years in middle-aged and older adults in the United States. Brain Sci. 2020;10(4):209. doi: 10.3390/brainsci10040209 32252391 PMC7225993

[100] Axon DR, Chien J. Predictors of mental health status among older United States adults with pain. Behav Sci (Basel). 2021;11(2):23. doi: 10.3390/bs11020023 33562841 PMC7914608

[101] Livingston G, Huntley J, Liu KY, Costafreda SG, Selbæk G, Alladi S, et al. Dementia prevention, intervention, and care: 2024 report of the Lancet standing Commission. Lancet. 2024;404(10452):572–628. doi: 10.1016/S0140-6736(24)01296-0 39096926

[102] Prajapati P, Barnard M, Bentley JP, Bhattacharya K. All-cause healthcare utilization among older adults with type 2 diabetes and cardiovascular and mental health comorbidities. BMC Geriatr. 2026;26(1):449. doi: 10.1186/s12877-026-06988-9 41618199 PMC13041125

[103] Castro-de-Araujo LFS, Cortes F, de Siqueira Filha NT, Rodrigues E da S, Machado DB, de Araujo JAP, et al. Patterns of multimorbidity and some psychiatric disorders: A systematic review of the literature. Front Psychol. 2022;13:940978. doi: 10.3389/fpsyg.2022.940978 36186392 PMC9524392

[104] Halstead S, Cao C, Høgnason Mohr G, Ebdrup BH, Pillinger T, McCutcheon RA, et al. Prevalence of multimorbidity in people with and without severe mental illness: A systematic review and meta-analysis. Lancet Psychiatry. 2024;11(6):431–42. doi: 10.1016/S2215-0366(24)00091-9 38642560

[105] Babey SH, Pourat N, Chen X, Lu C, Zhou W, O’Masta B, et al. The concurrent burden of multimorbidity, mental health conditions, and severe obesity among United States health center patients. Obesity Medicine. 2021;24:100348. doi: 10.1016/j.obmed.2021.100348

[106] Hudson JL, Chilcot J. Psychological Distress in Physical Long-Term Conditions. In: Goldsmith D, Covic A, Spaak J. Cardio-Renal Clinical Challenges. Cham: Springer International Publishing; 2015. p. 227–34.

[107] Shih M, Simon PA. Health-related quality of life among adults with serious psychological distress and chronic medical conditions. Qual Life Res. 2008;17(4):521–8. doi: 10.1007/s11136-008-9330-9 18365766

[108] Byles JE, Robinson I, Banks E, Gibson R, Leigh L, Rodgers B, et al. Psychological distress and comorbid physical conditions: Disease or disability?. Depress Anxiety. 2014;31(6):524–32. doi: 10.1002/da.22162 23922120

[109] Frise S, Steingart A, Sloan M, Cotterchio M, Kreiger N. Psychiatric disorders and use of mental health services by Ontario women. Can J Psychiatry. 2002;47(9):849–56. doi: 10.1177/070674370204700906 12500755

[110] Yang KH, Han BH, Moore AA, Palamar JJ. Trends in major depressive episodes and mental health treatment among older adults in the United States, 2010-2019. J Affect Disord. 2022;318:299–303. doi: 10.1016/j.jad.2022.09.007 36096373 PMC9530028

[111] Adepoju O, Lin S-H, Mileski M, Kruse CS, Mask A. Mental health status and healthcare utilization among community dwelling older adults. J Ment Health. 2018;27(6):511–9. doi: 10.1080/09638237.2018.1466030 29701495

[112] Blais RK, Tsai J, Southwick SM, Pietrzak RH. Barriers and facilitators related to mental health care use among older veterans in the United States. Psychiatr Serv. 2015;66(5):500–6. doi: 10.1176/appi.ps.201300469 25639990

[113] Wallerblad A, Möller J, Forsell Y. Care-Seeking pattern among persons with depression and anxiety: A population-based study in Sweden. Int J Family Med. 2012;2012:895425. doi: 10.1155/2012/895425 22655197 PMC3357962

[114] Essau CA. Frequency and patterns of mental health services utilization among adolescents with anxiety and depressive disorders. Depress Anxiety. 2005;22(3):130–7. doi: 10.1002/da.20115 16175563

