# Supplementary material for: Mental health care utilization among middle-aged and older U.S. adults: Findings from the National Health Interview Survey
Source: PLOS Ment Health. 2026 Oct 9;3(10):e0000744. doi: 10.1371/journal.pmen.0000744 (PMC13652341; doi:10.1371/journal.pmen.0000744)
Supplement: Supplementary file 1 [file pmen.0000744.s001.docx]

**Multicollinearity assessment of covariates**

The multicollinearity assessment showed **no evidence of problematic multicollinearity among the covariates**. The GVIF values were low for most variables, ranging from 1.048 for sex to 3.987 for income level, while the adjusted GVIF values, **GVIF^(1/2Df)^**, ranged from 1.024 to 1.971. Although income index (GVIF = 3.883; GVIF^(1/2Df)^ = 1.971) and income level (GVIF = 3.987; **GVIF^(1/2Df)^** = 1.259) had the highest values, these remained well below commonly used thresholds indicating concern. Depression (GVIF = 1.226; adjusted GVIF = 1.107) and anxiety (GVIF = 1.214; adjusted GVIF = 1.102) also showed minimal collinearity with the other covariates (see **S1 Table)**.

**S1 Table:**  **Collinearity diagnostics for predictors**

| **Variable** | **GVIF** | **Df** | **GVIF^(1/2Df)^** |
| --- | --- | --- | --- |
| Sex | 1.048 | 1 | 1.024 |
| Age group | 1.477 | 3 | 1.067 |
| Race | 1.37 | 6 | 1.027 |
| Education Category | 1.474 | 3 | 1.067 |
| Marital status | 1.203 | 2 | 1.047 |
| Urban-rural residence | 1.23 | 3 | 1.035 |
| Region | 1.18 | 3 | 1.028 |
| Comorbidity category | 1.34 | 2 | 1.076 |
| Smoking status | 1.134 | 2 | 1.032 |
| BMI | 1.153 | 3 | 1.024 |
| Income index | 3.883 | 1 | 1.971 |
| Income level | 3.987 | 3 | 1.259 |
| Insurance coverage | 1.063 | 1 | 1.031 |
| Depression | 1.226 | 1 | 1.107 |
| Anxiety | 1.214 | 1 | 1.102 |

GVIF, generalized variance inflation factor; Df, degrees of freedom; BMI, body mass index; CI, confidence interval; COR, crude odds ratio; AOR, adjusted odds ratio; GVIF^(1/2Df)^, adjusted generalized variance inflation factor.
