# Supplementary material for: Mental health care utilization among middle-aged and older U.S. adults: Findings from the National Health Interview Survey
Source: PLOS Ment Health. 2026 Oct 9;3(10):e0000744. doi: 10.1371/journal.pmen.0000744 (PMC13652341; doi:10.1371/journal.pmen.0000744)
Supplement: Supplementary file 2 [file pmen.0000744.s002.docx]

**Survey-weighted characteristics by MHC utilization status**

Similar to the unweighted sample, the survey-weighted distribution (see **S2 Table**) showed consistent trends in MHC utilization across most sociodemographic, socioeconomic, and health-related characteristics, with only slight differences in magnitude. Participants who utilized MHC services differed significantly from those who did not across all sociodemographic, socioeconomic, and health-related characteristics (p < 0.001). MHC users were more often female (65.5% vs. 48.8%; p < 0.001), had a lower median age (58.0 vs. 59.0 years; p < 0.001), and were more likely to be non-Hispanic White (78.1% vs. 64.2%; p < 0.001). They were also more likely to have some college/associate education (15.1% vs. 13.5%; p < 0.001), be neither married nor living with a partner (38.7% vs. 30.5%; p < 0.001), and reside in the Midwest (23.4% vs. 20.2%) or non-metropolitan areas (17.2% vs. 14.8%; p < 0.001). Socioeconomically, MHC users had a lower median family income-to-poverty ratio (3.3 vs. 3.7; p < 0.001) and were more likely to live below the poverty line (11.7% vs. 8.0%) or have low income (18.6% vs. 16.7%; p < 0.001). They were also more likely to be insured (96.6% vs. 92.1%; p < 0.001). Regarding health characteristics, MHC users had a greater comorbidity burden, with 52.2% having two or more comorbidities compared with 34.9% among non-users (p < 0.001). They were also more likely to smoke on some days (3.1% vs. 2.4%) or every day (13.8% vs. 9.1%; p < 0.001), and to be obese (41.0% vs. 33.2%; p < 0.001). Most notably, depression and anxiety were substantially more prevalent among MHC users than non-users (29.2% vs. 4.1% and 53.2% vs. 15.1%, respectively; both p < 0.001).

**S2 Table: Description of the weighted sample MHC utilization among middle-aged and older adults in the U.S**

| **Variables** | **Overall N = 156,597,525**  **100%%** | **Yes N = 32,835,701**  **20.97%** | **No N = 123,761,823**  **79.03%** | **p-value** |
| --- | --- | --- | --- | --- |
| **Sociodemographic characteristics** | | | | |
| **Sex^Φ^** |  |  |  | <0.001 |
| Male | 47.7 | 34.5 | 51.2 |  |
| Female | 52.3 | 65.5 | 48.8 |  |
| **Age in years^Φ^** | 59.0 (49.0, 69.0) | 58.0 (49.0, 68.0) | 59.0 (49.0, 69.0) | <0.001 |
| **Age category in years^Φ^** |  |  |  | <0.001 |
| 40-49 | 25.4 | 26.9 | 25 |  |
| 50-64 | 39 | 40.3 | 38.7 |  |
| 65-74 | 20.8 | 20 | 21 |  |
| ≥75 (75+) | 14.8 | 12.8 | 15.3 |  |
| **Race^Φ^** |  |  |  | <0.001 |
| Hispanic | 14.2 | 9.7 | 15.4 |  |
| Non-Hispanic White | 67.1 | 78.1 | 64.2 |  |
| Non-Hispanic Black | 10.9 | 8 | 11.7 |  |
| Non-Hispanic Asian | 5.7 | 2 | 6.7 |  |
| Non-Hispanic AIAN | 0.6 | 0.6 | 0.7 |  |
| Non-Hispanic AIAN, Multiple Races | 0.7 | 0.9 | 0.6 |  |
| Non-Hispanic Other/Multiple Races | 0.7 | 0.6 | 0.8 |  |
| **Educational category^τ^** |  |  |  | <0.001 |
| Less than high school | 11.9 | 10.2 | 12.3 |  |
| High school graduate/GED | 41.7 | 41.4 | 41.8 |  |
| Some college/Associate | 13.8 | 15.1 | 13.5 |  |
| Bachelor's or higher | 32.6 | 33.3 | 32.4 |  |
| **Marital status^τ^** |  |  |  | <0.001 |
| Married | 62.4 | 55.7 | 64.2 |  |
| Living with partner together as unmarried couple | 5.3 | 5.6 | 5.3 |  |
| Neither | 32.2 | 38.7 | 30.5 |  |
| **Region^τ^** |  |  |  | <0.001 |
| Northeast | 18 | 17.9 | 18.1 |  |
| Midwest | 20.8 | 23.4 | 20.2 |  |
| South | 38.1 | 37.9 | 38.2 |  |
| West | 23 | 20.8 | 23.5 |  |
| **Urban-rural residence^τ^** |  |  |  | <0.001 |
| Large Metro | 29 | 26.2 | 29.8 |  |
| Large Fringe Metro | 25.3 | 24.5 | 25.6 |  |
| Medium and Small Metro | 30.3 | 32.1 | 29.8 |  |
| Non-metropolitan | 15.3 | 17.2 | 14.8 |  |
| ***Socioeconomic characteristics*** | | | | |
| **Ratio of family income to poverty threshold^τ^** | 3.6 (2.0, 6.0) | 3.3 (1.7, 5.7) | 3.7 (2.0, 6.1) | <0.001 |
| **Income level^τ^** |  |  |  | <0.001 |
| Below poverty | 8.8 | 11.7 | 8 |  |
| Low income | 17.1 | 18.6 | 16.7 |  |
| Moderate | 29.4 | 28.2 | 29.7 |  |
| **Insurance coverage^δ^** |  |  |  | <0.001 |
| Uninsured | 7 | 3.4 | 7.9 |  |
| Insured | 93 | 96.6 | 92.1 |  |
| ***Behavioral and health characteristics*** | | | | |
| **Comorbidity category^τ^** |  |  |  | <0.001 |
| No comorbid | 32.1 | 20.9 | 35.1 |  |
| One comorbid | 29.4 | 26.9 | 30 |  |
| Two or more comorbid | 38.5 | 52.2 | 34.9 |  |
| **Smoking status^Φ^** |  |  |  | <0.001 |
| Not at all | 87.4 | 83.1 | 88.5 |  |
| Somedays | 2.6 | 3.1 | 2.4 |  |
| Everyday | 10.1 | 13.8 | 9.1 |  |
| **Body Mass Index (BMI) ^Φ^** |  |  |  | <0.001 |
| Underweight | 1.2 | 1.3 | 1.2 |  |
| Healthy weight | 27.6 | 25.2 | 28.2 |  |
| Overweight | 36.3 | 32.5 | 37.3 |  |
| Obese | 34.9 | 41 | 33.2 |  |
| **Depression^τ^** |  |  |  | <0.001 |
| No | 90.6 | 70.8 | 95.9 |  |
| Yes | 9.4 | 29.2 | 4.1 |  |
| **Anxiety^τ^** |  |  |  | <0.001 |
| No | 76.9 | 46.8 | 84.9 |  |
| Yes | 23.1 | 53.2 | 15.1 |  |

**^Φ^Predisposing factors** (Sex, age, race/ethnicity, marital status, smoking status, BMI.);**^δ^ Enabling factors** (Education, ratio of family income to poverty threshold, income level, insurance coverage, urban-rural residence, region);**^τ^ Need factors (**Comorbidity, depression, anxiety) based on Andersen’s Model of Health Utilization; %; Median (Q1, Q3); Pearson’s chi-squared (X^2^): Rao & Scott adjustment; Design-based KruskalWallis test; AIAN: American Indian and Alaska Natives.
