# Supplementary material for: Mental health care utilization among middle-aged and older U.S. adults: Findings from the National Health Interview Survey
Source: PLOS Ment Health. 2026 Oct 9;3(10):e0000744. doi: 10.1371/journal.pmen.0000744 (PMC13652341; doi:10.1371/journal.pmen.0000744)
Supplement: Supplementary file 3 [file pmen.0000744.s003.docx]

**Secondary logistic regression model without depression and anxiety**

**S3 Table** presents the secondary model with adjustment for depression and anxiety. The overall pattern was consistent with the primary model across all variables. In the univariate analysis, female ageing adults had higher odds of MHC utilization than males (COR = 1.99, 95% CI: 1.92–2.07; p < 0.001). Compared with adults aged 40–49 years, those aged 65–74 and ≥75 years had lower odds of MHC utilization (COR = 0.89, 95% CI: 0.84–0.94; and COR = 0.77, 95% CI: 0.73–0.82; both p *<* 0.001). Compared with Hispanic adults, non-Hispanic White and non-Hispanic AIAN adults with multiple races had higher odds, whereas non-Hispanic Asian adults had lower odds of MHC utilization (COR = 1.94, 95% CI: 1.83–2.06; COR = 2.53, 95% CI: 2.12–3.03; and COR = 0.48, 95% CI: 0.42–0.55; all p < 0.001). Compared with married adults, those living with an unmarried partner and those who were neither married nor partnered had higher odds of MHC utilization (COR = 1.23, 95% CI: 1.13–1.34; and COR = 1.47, 95% CI: 1.42–1.52; both p < 0.001). MHC utilization was also higher among adults residing in large fringe metropolitan, medium/small metropolitan, and non-metropolitan areas compared with large metropolitan areas. Compared with the Northeast, those in the Midwest had higher odds (COR = 1.18, 95% CI: 1.10–1.26; p < 0.001), whereas those in the West had lower odds (COR = 0.89, 95% CI: 0.83–0.97; p = 0.006). Adults with one or two or more comorbidities had higher odds than those without comorbidities (COR = 1.50 and 2.51, respectively; both p < 0.001). Compared with non-smokers, those who smoked on some days or every day had higher odds (COR = 1.37 and 1.62, respectively; both p < 0.001). Obese adults had higher odds of MHC utilization than those of normal weight (COR = 1.38, 95% CI: 1.32–1.44; p < 0.001). Compared with high-income adults, those living below poverty and those with low income had higher odds (COR = 1.59 and 1.21, respectively; both p < 0.001). Insured adults also had higher odds than uninsured adults (COR = 2.43, 95% CI: 2.22–2.66; p < 0.001).

After multivariable adjustment, female adults continued to have higher odds of MHC utilization than males (AOR = 2.08, 95% CI: 2.00–2.16; p < 0.001), while odds were lower among adults aged 50–64, 65–74, and ≥75 years than among those aged 40–49 years (AOR = 0.72, 0.48, and 0.36, respectively; all p < 0.001). Compared with Hispanic adults, non-Hispanic White adults had higher odds, whereas non-Hispanic Black and Asian adults had lower odds of MHC utilization (AOR = 1.90, 95% CI: 1.78–2.04; AOR = 0.79, 95% CI: 0.72–0.86; and AOR = 0.51, 95% CI: 0.45–0.59; all p < 0.001). Non-Hispanic AIAN adults with multiple races also had higher odds (AOR = 1.82, 95% CI: 1.49–2.22; p < 0.001). Compared with adults with a bachelor’s degree or higher, those with less than high school education, a high school diploma/GED, or some college/associate education had lower odds of MHC utilization (AOR = 0.71, 0.77, and 0.86, respectively; all p < 0.001). Compared with married adults, those living with an unmarried partner and those who were neither married nor partnered had higher odds (AOR = 1.13, 95% CI: 1.03–1.23; p = 0.007; and AOR = 1.31, 95% CI: 1.26–1.36; p < 0.001). Adults with one or two or more comorbidities had higher odds than those without comorbidities (AOR = 1.59 and 2.90, respectively; both p < 0.001). Compared with non-smokers, adults who smoked on some days or every day had higher odds (AOR = 1.35 and 1.37, respectively; both p < 0.001). Obesity was associated with higher odds of MHC utilization compared with normal weight (AOR = 1.18, 95% CI: 1.12–1.23; p < 0.001). Compared with high-income adults, those living below poverty, with low income, and with moderate income had higher odds (AOR = 1.70, 1.33, and 1.08, respectively; all p ≤ 0.001). Finally, insured adults had substantially higher odds of MHC utilization than uninsured adults (AOR = 2.38, 95% CI: 2.15–2.64; p < 0.001); see **S3 Table**.

**S 3 Table: Univariate and multiple logistic regression of factors associated with MHC utilization, excluding depression and anxiety**

| **Variable** | **Univariate Logistic Regression** | | | **Multivariable Logistic Regression** | | |
| --- | --- | --- | --- | --- | --- | --- |
| *Model statistics* | COR | 95%CI | p-value | AOR | 95%CI | p-value |
| **Sex^Φ^** |  |  |  |  |  |  |
| Male | Ref |  |  | Ref |  |  |
| Female | 1.99 | 1.92 – 2.07 | <0.001 | 2.08 | 2.00 – 2.16 | <0.001 |
| **Age category in years^Φ^** |  |  |  |  |  |  |
| 40-49 | Ref |  |  | Ref |  |  |
| 50-64 | 0.97 | 0.93 – 1.01 | 0.158 | 0.72 | 0.68 – 0.75 | <0.001 |
| 65-74 | 0.89 | 0.84 – 0.94 | <0.001 | 0.48 | 0.45 – 0.51 | <0.001 |
| ≥75 (75+) | 0.77 | 0.73 – 0.82 | <0.001 | 0.36 | 0.33 – 0.38 | <0.001 |
| **Race^Φ^** |  |  |  |  |  |  |
| Hispanic | Ref |  |  | Ref |  |  |
| Non-Hispanic White | 1.94 | 1.83 – 2.06 | <0.001 | 1.9 | 1.78 – 2.04 | <0.001 |
| Non-Hispanic Black | 1.09 | 0.99 – 1.19 | 0.071 | 0.79 | 0.72 – 0.86 | <0.001 |
| Non-Hispanic Asian | 0.48 | 0.42 – 0.55 | <0.001 | 0.51 | 0.45 – 0.59 | <0.001 |
| Non-Hispanic AIAN | 1.54 | 1.05 – 2.26 | 0.029 | 1.12 | 0.70 – 1.77 | 0.642 |
| Non-Hispanic AIAN, Multiple Races | 2.53 | 2.12 – 3.03 | <0.001 | 1.82 | 1.49 – 2.22 | <0.001 |
| Non-Hispanic Other/Multiple Races | 1.27 | 1.00 – 1.60 | 0.048 | 1.08 | 0.86 – 1.37 | 0.504 |
| **Educational category^δ^** |  |  |  |  |  |  |
| Bachelor's or higher | Ref |  |  | Ref |  |  |
| Less than high school | 0.81 | 0.76 – 0.86 | <0.001 | 0.71 | 0.66 – 0.77 | <0.001 |
| High school graduate/GED | 0.96 | 0.93 – 1.00 | 0.073 | 0.77 | 0.74 – 0.81 | <0.001 |
| Some college/Associate | 1.09 | 1.03 – 1.14 | 0.001 | 0.86 | 0.81 – 0.91 | <0.001 |
| **Marital status^δ^** |  |  |  |  |  |  |
| Married | Ref |  |  | Ref |  |  |
| Living with partner together as unmarried couple | 1.23 | 1.13 – 1.34 | <0.001 | 1.13 | 1.03 – 1.23 | 0.007 |
| Neither | 1.47 | 1.42 – 1.52 | <0.001 | 1.31 | 1.26 – 1.36 | <0.001 |
| **Urban-rural residence^δ^** | |  |  |  |  |  |
| Large Metro | Ref |  |  | Ref |  |  |
| Large Fringe Metro | 1.09 | 1.03 – 1.15 | 0.004 | 0.95 | 0.90 – 1.00 | 0.06 |
| Medium and Small Metro | 1.22 | 1.15 – 1.30 | <0.001 | 0.97 | 0.92 – 1.03 | 0.307 |
| Non-metropolitan | 1.31 | 1.22 – 1.41 | <0.001 | 0.93 | 0.87 – 1.00 | 0.037 |
| **Region^δ^** |  |  |  |  |  |  |
| Northeast | Ref |  |  | Ref |  |  |
| Midwest | 1.18 | 1.10 – 1.26 | <0.001 | 1.06 | 0.99 – 1.13 | 0.093 |
| South | 1 | 0.94 – 1.08 | 0.927 | 1.02 | 0.96 – 1.08 | 0.552 |
| West | 0.89 | 0.83 – 0.97 | 0.006 | 0.99 | 0.92 – 1.06 | 0.728 |
| **Comorbidity category^τ^** | |  |  |  |  |  |
| No comorbid | Ref |  |  | Ref |  |  |
| One comorbid | 1.5 | 1.43 – 1.57 | <0.001 | 1.59 | 1.51 – 1.67 | <0.001 |
| Two or more comorbid | 2.51 | 2.40 – 2.63 | <0.001 | 2.9 | 2.76 – 3.05 | <0.001 |
| **Smoking status^Φ^** | |  |  |  |  |  |
| Not at all | Ref |  |  | Ref |  |  |
| Somedays | 1.37 | 1.25 – 1.50 | <0.001 | 1.35 | 1.23 – 1.49 | <0.001 |
| Everyday | 1.62 | 1.53 – 1.70 | <0.001 | 1.37 | 1.29 – 1.45 | <0.001 |
| **Body Mass Index (BMI) ^δ^** | |  |  |  |  |  |
| Normal | Ref |  |  | Ref |  |  |
| Underweight | 1.14 | 1.00 – 1.31 | 0.054 | 0.96 | 0.82 – 1.11 | 0.553 |
| Overweight | 0.97 | 0.94 – 1.01 | 0.201 | 1.03 | 0.99 – 1.08 | 0.136 |
| Obese | 1.38 | 1.32 – 1.44 | <0.001 | 1.18 | 1.12 – 1.23 | <0.001 |
| **Income level^δ^** | |  |  |  |  |  |
| High | Ref |  |  | Ref |  |  |
| Below poverty | 1.59 | 1.50 – 1.68 | <0.001 | 1.7 | 1.59 – 1.81 | <0.001 |
| Low income | 1.21 | 1.16 – 1.28 | <0.001 | 1.33 | 1.26 – 1.41 | <0.001 |
| Moderate | 1.04 | 1.00 – 1.08 | 0.086 | 1.08 | 1.03 – 1.12 | 0.001 |
| **Insurance coverage^δ^** |  |  |  |  |  |  |
| Uninsured | Ref |  |  | Ref |  |  |
| Insured | 2.43 | 2.22 – 2.66 | <0.001 | 2.38 | 2.15 – 2.64 | <0.001 |

**Observations:** 121,032**; ^Φ^Predisposing factors** (Sex, age, race/ethnicity, marital status, smoking status, BMI);**^δ^ Enabling factors** (Education, ratio of family income to poverty threshold, income level, insurance coverage, urban-rural residence, region);**^τ^ Need factors (**Comorbidity, depression, anxiety) based on Andersen’s Model of Health Utilization; Univariate logistic regression analyses were initially conducted to examine the association between each predictor and MHC utilization. Variables demonstrating a statistically significant overall association with MHC utilization (p ≤ 0.05) were selected for inclusion in the multivariable logistic regression model. For categorical predictors selected for multivariable analysis, all constituent categories were retained in the model regardless of the statistical significance of individual category-level coefficients, with one category specified as the reference group. This approach ensured that categorical predictors were evaluated as complete variables and avoided selective exclusion of individual categories based solely on their category-specific p-values. The multivariable model accounted for potential confounding and estimated adjusted associations between the predictors and MHC utilization.
